# A Data-Driven Perspective on Bioisostere Evaluation: Mapping the Benzene Bioisostere Landscape with BioSTAR

**DOI:** 10.1021/acs.jmedchem.5c01641

**Published:** 2025-08-05

**Authors:** Pol Hernández-Lladó, Nicholas A. Meanwell, Angela J. Russell

**Affiliations:** † Department of Chemistry, Chemistry Research Laboratory, 6396 University of Oxford, Mansfield Road, Oxford OX1 3TA, U.K.; ‡ Department of Pharmacology, University of Oxford, Mansfield Road, Oxford OX1 3QT, U.K.; § 480639 The Baruch S. Blumberg Institute, 3805 Old Easton Road, Doylestown, Pennsylvania 18902, United States; ∥ The School of Pharmacy, The University of Michigan, Ann Arbor, Michigan 48109, United States; ⊥ The Ernest Mario School of Pharmacy, Rutgers University, Piscataway, New Jersey 08854, United States; # NuArq MedChem Consulting LLC, Yardley, Pennsylvania 19067, United States

## Abstract

The bioisostere landscape is continually expanding, with new scaffolds emerging as alternatives in drug design. Increasingly, medicinal chemists face the challenge of selecting and prioritising these bioisosteres, often relying on personal experience and anecdotal evidence. In this Perspective, we lay out a data-driven approach to analyze the bioisostere landscape, using benzene bioisosteres as a representative example, and quantitatively compare replacements based on their impact on bioactivity, solubility, and metabolic stability. To support the findings of the analysis, we highlight recent and particularly elegant examples of benzene bioisostere applications while identifying areas where further development could significantly benefit the community. By providing this Perspective and associated data-mining workflow (BioSTAR), we aim to support more informed decision-making in bioisosteric replacement selection in drug design and inspire future innovations in bioisostere design.

## Graphical abstract

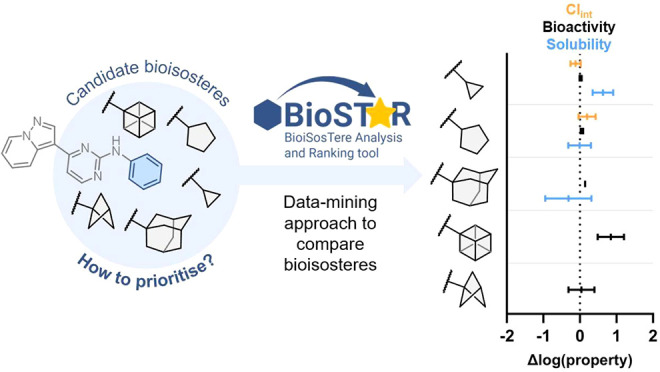

## Significance

- The expanding bioisostere landscape poses a central challenge in medicinal chemistry: how to systematically compare and prioritize scaffolds.
- This Perspective advocates for a data-driven approach and provides a data-mining workflow (BioSTAR) to evaluate bioisosteric replacements.
- Using benzene bioisosteres as a case study, we assess their impact on bioactivity, solubility, and metabolic stability.
- The workflow serves as a practical tool for medicinal chemists and a foundation for collaborative, data-driven progress in molecular design.

## Introduction

Over the past two decades, particularly following the landmark publication of the “Escape from Flatland” paper by Lovering et al.,^1^ the medicinal chemistry community has sought to increase the three-dimensionality of lead compounds. This is because a higher Fsp^3^ correlates with better progression from discovery through preclinical and clinical development and to the market, as well as improved physicochemical and absorption, distribution, metabolism and elimination (ADME) properties.^1^ Fsp^3^, as well as number of chiral centers, is also linked to decreased promiscuity, and thus may help address toxicity, a leading cause of attrition in the clinic.^2^ More recent analyses suggest that the utility of Fsp^3^ as a predictive metric for clinical trial progression may have been overstated, as the trends observed in 2009 have not persisted.^3^ Notwithstanding, other metrics related to compound three-dimensionality also correlate with improved developability. For example, Ritchie and Macdonald elegantly showed that an increased aromatic ring count correlates with lower solubility, higher serum albumin binding, and higher clogP, leading to overall poorer developability.^4^These results align with Leeson’s findings, which highlight reduced carboaromaticity as a key feature distinguishing approved drugs from other molecules acting on the same target.^5^ In line with these analyses, a common strategy in medicinal chemistry to improve molecular properties has been the use of arene bioisosteres to replace sp^2^-rich aromatic rings with sp^3^-rich alternative motifs. This has led to the development and rediscovery of scaffolds designed to serve as arene bioisosteres.

The growing interest in this area is evident from the surging number of publications referencing the term ″bioisostere″ since 2009. Caged hydrocarbons have received particular attention, as exemplified by bicyclo[1.1.1]­pentanes (BCPs),^6–8^ bicyclo[2.1.1]­hexanes (BCHs),^9–11^ bicyclo[3.1.1]­heptanes (BCHeps),^12,13^ cubanes,^14–16^ and other, less common scaffolds such as cuneanes^17–20^ and stellanes.^21^ Their popularity can be attributed to their well-defined exit vectors, which can closely mimic those of arenes, along with their high sp^3^ character and rigidity, which minimizes entropic penalties upon target binding. Several excellent reviews have examined the benzene bioisostere field.^22–26^

Medicinal chemists are increasingly incorporating these scaffolds into their compound designs. By replacing a benzene ring with a potentially bioisosteric group, the designer aims to improve a molecule’s overall profile, for example, increasing its solubility or metabolic stability, while preserving bioactivity. Nonetheless, the impact of such replacements on bioactivity and other molecular properties is often context-dependent, making their impact challenging to predict. Compounding this, the growing number of benzene bioisosteres in the literature raises a new challenge: selecting which isosteric scaffolds to prioritise for synthesis.

In this perspective, we have employed a data-driven approach to evaluate and compare benzene bioisosteres in order to address these challenges. To achieve this, we developed a data-mining workflow, which we have made publicly available and offers a versatile, user-friendly framework for evaluating other bioisosteric replacements.

## Bioisosteres: Definition and Evaluation

### Isostere and Bioisostere Definitions

The concept of isosterism was first introduced by Irving Langmuir in 1919,^27^ building on earlier work by James Moir in 1909.^28^ Initially, the definition was restricted to pairs of molecules or atomic groups with similar electron configurations, such as N_2_O and CO_2_. In 1932, Erlenmeyer broadened this definition to include ″*elements, molecules, or ions in which the peripheral layers of electrons may be considered identical*.″^29^

The term bioisosterism was introduced by Friedman in 1950, who defined bioisosteres as structural moieties that ″*fit the broadest definition of isosteres and exhibit the same type of biological activity*.″^30^ In this seminal work, Friedman emphasized that while isosterism was a necessary condition for bioisosterism, it was not sufficient due to the complexity of biological systems and the characteristics of molecular recognition. The publication also presented an early example of matched molecular pair (MMP) analysis.

Thornber later expanded Friedman’s definition to include nonclassical bioisosteres, describing bioisosteres as ″groups or molecules with chemical and physical similarities that result in broadly similar biological properties.″^31^ This expanded definition is widely accepted today.

### Data-Mining Approaches for Bioisostere Evaluation

Potential bioisosteric replacements can be evaluated through data-mining methods that identify MMPs in the literature and analyze the effects of the replacements on properties such as bioactivity, solubility, or metabolic stability.^32,33^

EMIL, the first database of this kind manually curated by Fujita and colleagues in the early 1990s, collated examples of MMPs and summarized the effect the molecular scaffold had on activity, toxicity, metabolic stability and other parameters.^34^ BIOSTER, developed at the same time by Ujváry, is a similar database containing over 28,000 bioisosteric transformations manually curated from the literature, although patents are excluded.^35^

*In silico* methods were later developed to perform comprehensive analyses of the literature. These employ fragmentation and clustering algorithms to mine bioactivity databases and identify bioisosteric replacements. Sheridan and Miller’s maximum common substructure (MCS) algorithm,^36^ and Hussain and Rea’s fragmentation and indexing (F+I) algorithm are common methods employed to achieve this.^37^

An example of an *in silico*-curated bioisostere database is SwissBioisostere, a web-based, freely accessible resource that enables users to search for bioisosteric replacements of specific scaffolds and provides a summary of their effects on activity, Log*P*, topological polar surface area (tPSA), and molecular weight.^38,39^ SwissBioisostere uses data from the ChEMBL database, processed through a fragmentation and indexing algorithm, and is a powerful, user-friendly tool; however, it was last updated in 2021 (ChEMBL version: 28) and its algorithm is not open-access.

A complementary, *in silico*-curated tool, the Ring Replacement Recommender, was developed by Ertl et al.^40^ This web-based resource suggests alternative ring systems for the 245 most frequently used rings, prioritising those associated with at least a 2-fold increase in potency. The recommendations are derived from MMP analysis of data from ChEMBL (version 29).

Data-mining approaches have also been successfully applied to protein–ligand complexes. These are hybrid methods that compare interaction pattern graphs of published crystal structures and identify alternative molecular scaffolds capable of achieving similar interactions with the protein.^41^ sc-PDB-frag is an online tool that identifies bioisosteric replacements for specific fragments bound to a protein through mining of the protein data bank (PDB).^42^ The scarcity of cocrystal structures available limits the application of these methods to well-precedented scaffolds.

## An Open-Source Data-Mining Workflow for Bioisostere Evaluation

To evaluate and compare potential benzene bioisosteres, we quantified the overall effect of these molecular replacements on the key properties of bioactivity, solubility, metabolic stability, and membrane permeability. This analysis was conducted using an open-access data-mining workflow, which we termed BioSTAR (BioiSosTere Analysis and Ranking).^200^ The BioSTAR workflow employs free, open-source software and can be run on a single benchtop computer by users with no computational experience. It uses Knime as the data-processing software and ChEMBL (version 35, released December 2024) as the open-access database, but it can also be applied to other databases. The output files are visualized with the open-source program DataWarrior.^43^ These characteristics allow others to repeat the bioisostere evaluation over time and as more data becomes available, and apply it to any molecular transformation of interest.

Employing the BioSTAR workflow we identified MMPs in ChEMBL differing only by the bioisostere investigated and compared all of their available data (bioactivity, solubility, clearance and membrane permeability). Only homogeneous pairs, i.e., pairs of data points obtained from the same assay in the same publication, were included in the analysis. This was informed by the studies of Kramer and co-workers who showed that many fewer data points are required to reach statistical significance when using homogeneous pairs, when compared to a combination of homogeneous and heterogeneous pairs.^44^ This condition for homogeneity also allowed a more computationally efficient workflow, since only structures within the same document were included in the MMP-searching algorithm.

The data-mining workflow employed is summarized below and in Figure 1:

1. Structure preparation by removal of salts and stereocenters. The F+I algorithm employed in the workflow cannot handle defined stereocenters or double bond geometries, which is an important limitation. Nonetheless, the workflow output can be later processed to extract this information.
2. Substructure search in ChEMBL for the identification of molecules containing the scaffold of interest.
3. Extraction of documents containing the scaffold of interest.
4. Mining of all structures within the identified documents.
5. Mining of all data associated with these structures. Only exact values, without qualifiers such as < or >, were included in the analysis.
6. Processing of the resulting structures with a fragmentation algorithm. The fragmentation algorithm (Hussain and Rea)^37^ was applied for 1 and 2 cuts for acyclic single bonds to rings, although the selection of alternative fragmentation patterns is possible to explore other replacements. Filtering based on the number of heavy atoms in the investigated scaffolds was applied to streamline processing.
7. Filtering for homogeneous pairs. Only MMPs with molecules originating from the same document and data derived from the same assay were retained for analysis.
8. Calculation of differences in properties.
9. Substructure search to filter results by transformation.

**Figure 1:**
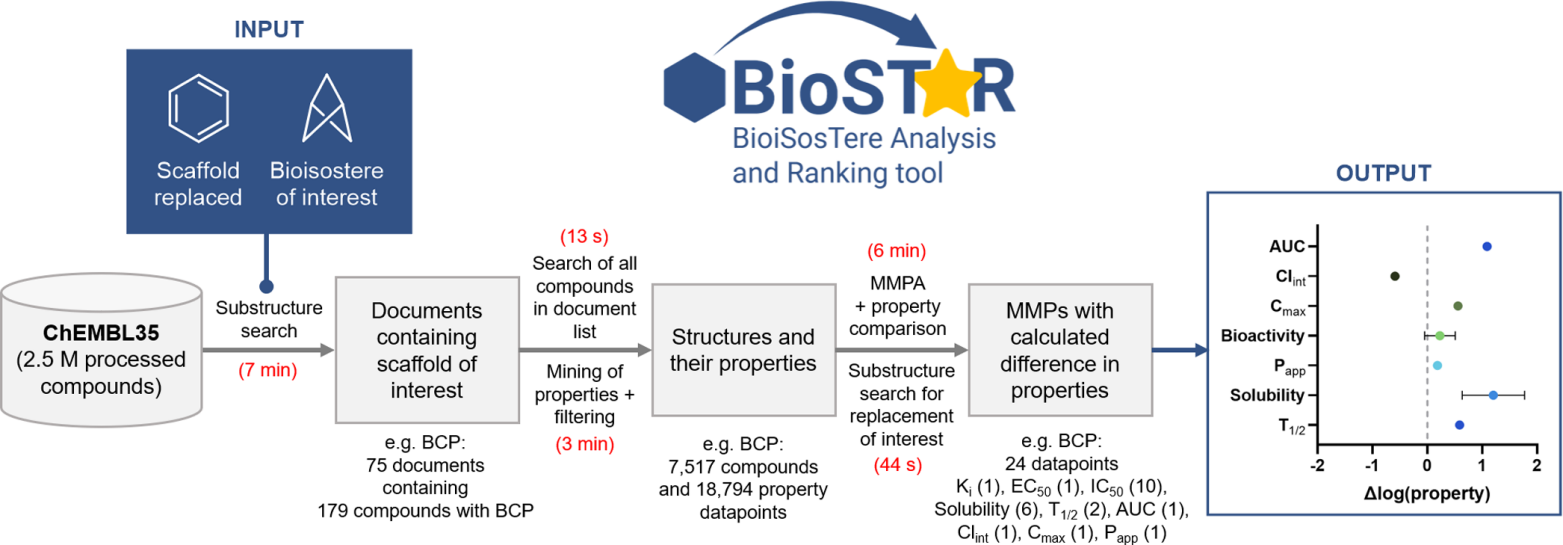
Schematic representation of the BioSTAR workflow and results obtained when performing search on BCP scaffold. In red, time required to complete each process.

The workflow described above allowed us to collate all the data available on ChEMBL for a list of scaffolds of interest. This process could be performed on a benchtop computer with no need for parallel processing or extended times. For example, the data-mining required to evaluate the replacement of a *para*-substituted benzene ring for a disubstituted BCP was completed in 16 min and 57 s.^45^ The BioSTAR workflow was designed for use by medicinal chemists with no previous computational experience, requiring as input solely the structure of the scaffold being replaced and the potential bioisostere investigated.

## Quantitative Evaluation of Benzene Bioisosteres

The BioSTAR workflow was used to evaluate and compare benzene bioisosteres. This was motivated by the increasing number of scaffolds described as such, which often leads to challenges in prioritisation of designed compounds for synthesis.

### Summary of the Matched Molecular Pair Analysis

We initiated the data-mining analysis by selecting the benzene bioisosteres to be investigated. The selection was guided by previous reports of bioisosterism from both the primary literature and review articles, with a focus on saturated mono- and polycyclic scaffolds.^22,23,25,26^ The selected scaffolds are compiled in Figure 2. Heteroaromatic ring systems, often used as benzene bioisosteres, were excluded from this analysis in favor of nonclassical bioisosteres. Nonetheless, the general applicability of the BioSTAR workflow allows it to be extended to the analysis of such substitutions. The role of heteroaromatics as benzene bioisosteres has previously been reviewed by Ritchie and Macdonald,^46^ and Subbaiah and Meanwell.^23^

**Figure 2:**
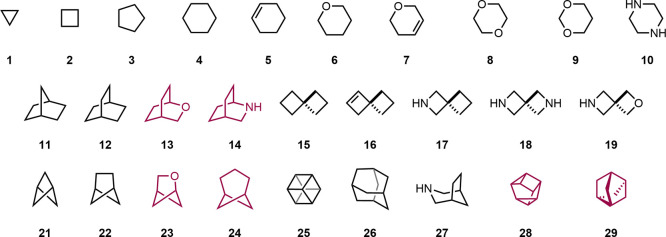
Scaffolds investigated as potential benzene bioisosteres. No homogeneous MMPs were found in ChEMBL (version 35) for the scaffolds colored red.

Using the BioSTAR workflow on the ChEMBL database (version 35), we extracted bioactivity data for 21,868 homogeneous MMPs containing the scaffolds shown in Figure 2. The molecular replacements with the highest number of homogeneous pairs were monosubstituted benzene ring to cyclohexane (*N* = 9672), cyclopropane (*N* = 3982), cyclopentane (*N* = 2551) or cyclobutene (*N* = 1075). For *para*-substituted benzene rings, replacement by a 1,4-cyclohexane was the most precedented substitution (*N* = 876), which was also true for *meta*- (1,3-cyclohexane, *N* = 112) and *ortho*-substituted benzenes (1,2-cyclohexane, *N* = 116). On the other hand, no MMPs were found containing oxabicyclo[2.2.2]­octane (**13**), azabicyclo[2.2.2]­octane (**14**), oxabicyclo[2.1.1]­hexane (**23**), BCHep (**24**), cuneane (**28**) or stellane (**29**).

The available data on physicochemical and ADME properties in ChEMBL was considerably more limited. For the bioisosteres investigated, we obtained a total of 202 solubility, 198 clearance, and 132 permeability data points. Similar to the bioactivity data, MMPs involving the replacement of a monosubstituted benzene with a cyclohexane ring were the most frequently observed across all three properties. At this time, for many of the investigated scaffolds no data beyond bioactivity was available.

A key limitation of this data-driven approach is the positive bias introduced by the under-reporting of unsuccessful experiments. This bias is likely more pronounced for less-precedented scaffolds, thus requiring cautious interpretation of the results obtained with these cases. Additionally, the partial coverage of the patent literature in ChEMBL is another important factor to consider when evaluating the outcomes of this analysis.

### Effects on Bioactivity

The change in bioactivity resulting from a molecular transformation defines its bioisosteric nature; consequently, analyzing this effect is central to evaluating bioisosteres. The results of the MMP analysis are summarized in Figure 3, which illustrates the impact that each molecular transformation had on bioactivity, solubility, and clearance. Figure 3 includes transformations with 5 or more data points in ChEMBL, but all of the results from the analysis, including descriptive statistics, can be found in the Supporting Information (Tables S1–S4).

**Figure 3:**
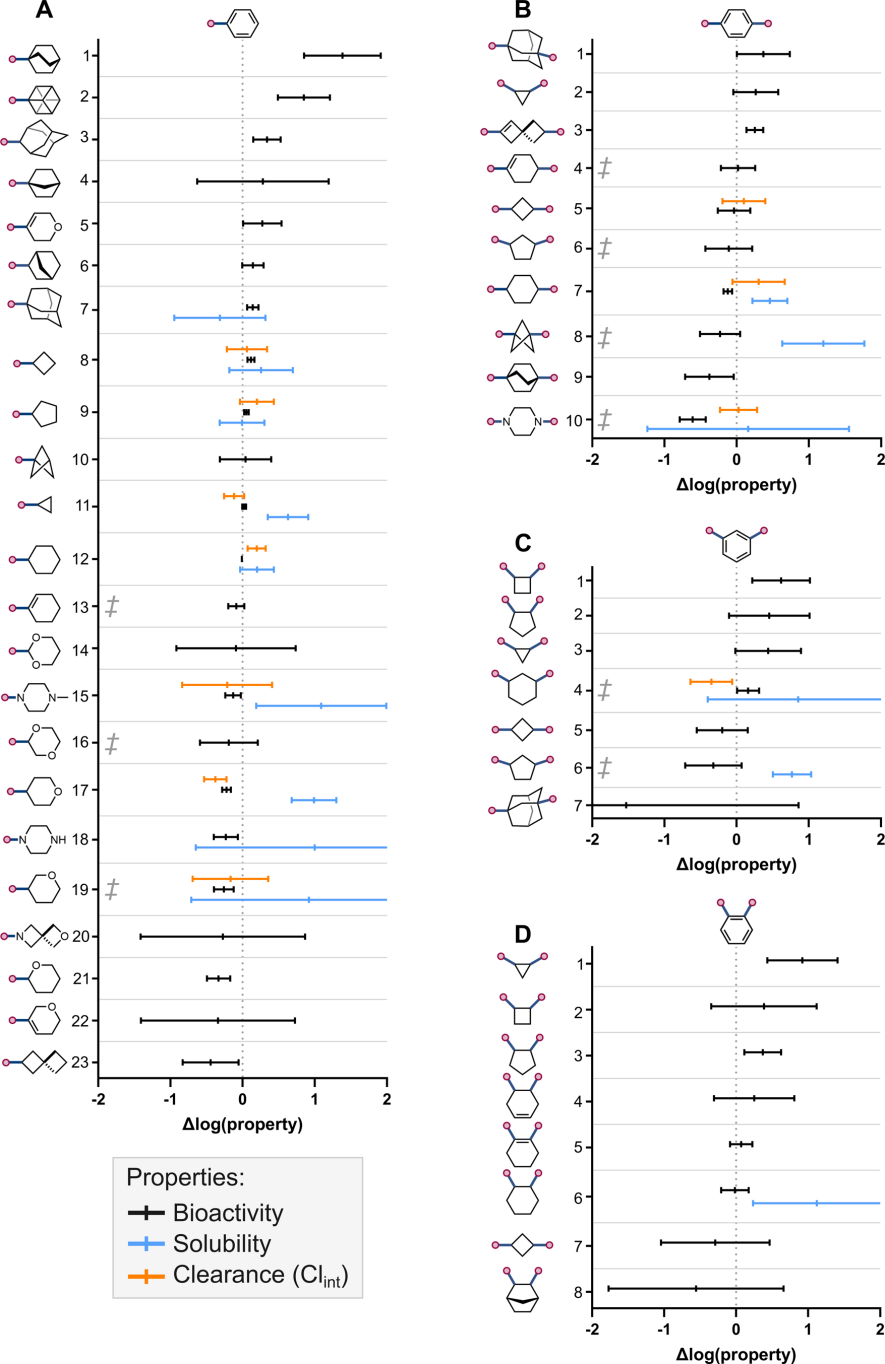
Summary of the impact of potentially bioisosteric replacements on bioactivity, solubility, and clearance (Cl_int_). Mean values are plotted with error bars representing 95% confidence intervals. (A) Terminal benzene; (B) para-substituted benzene; (C) meta-substituted benzene; (D) ortho-substituted benzene. ^‡^Indicates substitutions with nonsignificant context dependency (see Supporting Information, Tables S1–S7 for details).

A key aspect of bioisosterism is its inherent context dependency. Despite its significance, this consideration has not been explicitly incorporated into the analysis thus far. To evaluate the generality of the results obtained, we classified each bioactivity data point according to its biological target, based on ChEMBL’s protein classification (level 3). The effect of the molecular replacements on bioactivity (Δ­(−log­(bioactivity))) against each target family were measured. Subsequently, we employed a Welch’s one-way analysis of variance (ANOVA) to assess whether the target family influenced the observed Δ­(−log­(bioactivity)). A statistically significant result would indicate that the effect of the molecular replacement depended on the context of the target family, underscoring the context-dependent nature of bioisosterism. Conversely, a nonsignificant result would suggest that the target family did not have a significant impact on the observed effect, implying that the bioisostere can be considered more general in its applicability. This approach therefore allowed the assessment of the context-dependency of the investigated bioisosteric replacements. The transformations that showed no statistically significant context dependency are highlighted in Figure 3. Detailed results of this analysis are included in the Supporting Information (Tables S1–S4).

Among the scaffolds investigated as monosubstituted benzene bioisosteres, cyclopentane, BCP, cyclopropane, and cyclohexane showed the smallest effect on bioactivity (Figure 3A, rows 9–12). For cyclohexane, this was in line with Gunaydin and Bartberger’s analysis, which showed that 36–45% of terminal phenyl to cyclohexyl substitutions lead to ΔpIC_50_ ≤ 0.3 (≤2-fold change).^47^ In our analysis, this condition was true for 39% of the pairs adjudicated. Nonetheless, the bioisosteric nature of cyclohexane, cyclopentane, and cyclopropane was strongly context dependent, with effects on bioactivity varying across target families. A more general replacement based on our analysis, with similarly small effect on bioactivity, was 1-cyclohexene (row 13). Both bicyclo[2.2.2]­octane (BCO) and cubane showed significantly positive effects on bioactivity compared to benzene (Figure 3A, rows 1–2), which may be due to entropy-driven effects.

Figure 3B shows the results for *para*-substituted benzene rings. The replacements leading to smallest changes in bioactivity were 1,4-substituted cyclohexene, 1,3-substituted cyclobutane, 1,3-substituted cyclopentane, and 1,4-substituted cyclohexane (Figure 3B, rows 4–7). Among these, 1,4-substituted cyclohexene and 1,3-substituted cyclopentane showed nonsignificant effects of context, and thus were the most general replacements. The context-dependency of the replacement of a *para*-substituted benzene by 1,2-substituted cyclopentane could not be assessed due to the limited data available (2 target families). Based on our analysis, a more generally bioisosteric replacement of *para*-substituted benzene rings is the 1,3-substituted BCP moiety (Figure 3B, row 8).

The impact of potentially bioisosteric replacements for *meta*- and *ortho*-substituted benzene rings are summarized in Figure 3C,D. In both cases, 1,2- and 1,3-disubstituted monocycloalkanes emerged as the most frequently reported replacements.

For a *meta*-substituted benzene, 1,3-substituted cyclohexane and 1,3-substituted cyclobutane exhibited the smallest effects on bioactivity (Figure 3C, rows 4–5), with context having no significant impact on the effects derived from the former. Although less precedented, the use of 1,3-substituted cyclopentane as a *meta*-substituted benzene bioisostere was supported by our analysis, which showed that this replacement led to no significant impact on bioactivity across target families (Figure 3C, row 6).

For *ortho*-substituted benzene, 1,2-substituted cyclohexane and 1,2-substituted cyclohexene resulted in the smallest changes in bioactivity, although the observed effects were strongly context-dependent (Figure 3D, rows 5–6).

These findings underscore the scarcity of effective bioisosteric replacements for *meta*- and *ortho*-substituted benzene rings. Recent studies have highlighted the potential of 2-oxabicyclo[2.1.1]­hexanes,^48^ BCHeps,^13^ and 1,3-cubanes^14^ as *meta*-benzene bioisosteres, and of BCHs^49^ as *ortho*-benzene bioisosteres. Nonetheless, additional data are needed to further validate their bioisosteric behavior and impact on solubility, clearance, and other molecular properties. Developing further methods to synthesize appropriately substituted carbocyclic scaffolds, such as BCHs, BCHeps and cubanes, and incorporating these into bioactive molecules will be crucial to address this gap in the literature.

An important consideration when interpreting Figure 3 is the influence of lipophilicity on the effect of bioisosteric replacements. Increased lipophilicity often correlates with enhanced bioactivity, largely due to favorable desolvation and associated entropic gains during binding.^50^ Nonetheless, as noted by Hann, such potency gains may come at the expense of overall developability, due to poorer physicochemical properties of structures suffering from “molecular obesity”.^51^ In this data set, there was no overall correlation between Δlog­(bioactivity) and ΔclogP when aggregating all bioisosteric replacements, suggesting that lipophilicity is not the primary driver of the observed bioactivity changes (see Supporting Information, Figure S2 for details). However, when focusing on the replacement of *mono*-substituted benzene, a weak but statistically significant correlation was detected, indicating that lipophilicity is contributing to the bioactivity changes observed in this subset of transformations. No significant correlation between Δlog­(bioactivity) and ΔclogP was detected for the replacement of *para*-, *meta*-, or *ortho*-substituted benzene.

The BioSTAR workflow cannot handle stereocenters, and thus the aforementioned results did not distinguish between *cis* and *trans* isomers of disubstituted rings. The workflow outputs could, however, be analyzed based on the relative configuration of stereocenters (see Supporting Information, Figure S1 for details). The only replacement where the impact on bioactivity was significantly different between stereoisomers was the replacement of a *para*-substituted benzene ring with a 1,4-substituted cyclohexane. In this instance, *trans*-1,4-substituted cyclohexane was a better bioisostere than its *cis* stereoisomer, leading to a smaller change in bioactivity (Figure 4). The closer geometric similarity between the exit vectors of the *trans* isomer and the *para*-substituted benzene may account for this difference in behavior (Figure 4).

**Figure 4:**
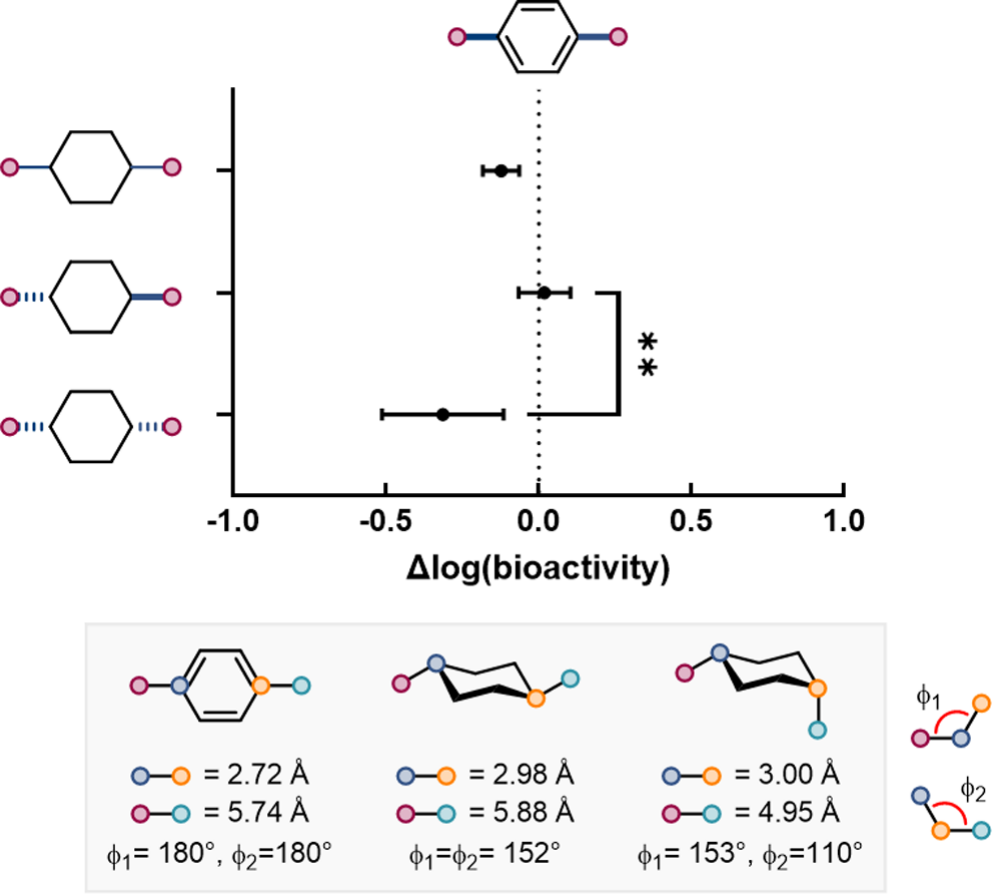
Effect of relative stereochemistry on 1,4-cyclohexanes as para-substituted benzene bioisosteres. Data are presented as mean ± 95% confidence interval. Welch’s *t* test was used to assess whether the mean bioactivity changes differed significantly between cis and trans isomers, accounting for unequal variances. Significance is indicated as follows: *p* < 0.05 (*), *p* < 0.01 (**). Summary of the exit vector geometries for 1,4-dimethylbenzene, trans- and cis-1,4-dimethylcyclohexane.

### Effects on Solubility

The bioisosteric replacement of benzene rings is often used in compound design to improve the physicochemical properties of the molecules, including their solubility. The BioSTAR workflow has allowed a comprehensive analysis of the literature to evaluate whether these replacements have a significant impact on solubility, as well as ADME properties such as metabolic stability and cell permeability.

Solubility measurements are highly condition-dependent; however, the data filtering process in the BioSTAR workflow, restricted to homogeneous pairs (i.e., data points from the same publication measured under identical conditions), mitigates this source of variability. The results of this solubility analysis, which includes kinetic and thermodynamic measures, are also included in Figure 3 (see Supporting Information, Table S5 for details). Significant increases in solubility were observed when a monosubstituted benzene was replaced with a cyclopropyl, an *N*-methyl piperazine, and a 4-tetrahydropyran (4-THP) ring (Figure 3A, rows 11, 15, 17). These improvements were likely due to reduced hydrophobicity and/or disrupted crystal packing.^52^ Indeed, a statistically significant correlation was detected between Δlog­(solubility) and ΔclogP, linking increases in solubility to decreased clogP (see Supporting Information, Figure S2 for details). Substitution of a phenyl ring by piperazine and 3-THP heterocycles also resulted in enhanced solubility, albeit not to a statistically significant extent (Figure 3A, rows 18–19). The replacement of a phenyl group with other hydrophobic moieties, such as adamantyl, cyclobutyl, cyclopentyl, and cyclohexyl resulted in no substantial changes in solubility, as is evident from the data presented in Figure 3A, rows 7–9 and 12.

Our analysis confirmed that the use of 1,4-substituted cyclohexane and BCP as *para*-substituted benzene bioisosteres leads to a statistically significant increase in solubility (Figure 3B, rows 7–8). Notably, the incorporation of BCP produced the largest effect in the data set, resulting in a mean solubility increase of over an order of magnitude. These findings were in line with earlier reports in the literature.^8,53^

Solubility data in ChEMBL for *meta*- and *ortho*-substituted benzene derivatives are limited. Nevertheless, significant solubility enhancements were observed when replacing a *meta*-substituted benzene with a 1,3-substituted cyclopentane (Figure 3C, row 6) and an *ortho*- substituted benzene with a 1,2-substituted cyclohexane (Figure 3D, row 6).

### Effects on Metabolic Stability

Benzene rings are susceptible to oxidative metabolism to give phenols and dihydroxybenzenes, with the latter subject to further oxidation to afford quinone-based species that can form glutathione adducts.^54^ In order to avoid this, and to improve the metabolic stability of compounds, practitioners often replace these aromatic rings with saturated bioisosteres. We employed the BioSTAR workflow to evaluate the effect these replacements had on metabolic stability across the ChEMBL database. Homogenous pairs of both hepatocyte and liver microsome clearance data were pooled, as well as *in vivo* measures of clearance. Despite these broad inclusion criteria, the clearance data available are rather scarce, which limited the number of replacements that could be evaluated. The results of this analysis are summarized in Figure 3 (see Supporting Information, Table S6 for details).

For monosubstituted benzene rings, the only replacement that led to a statistically significant reduction in clearance was the substitution of a phenyl group with a 4-THP heterocycle (Figure 3A, row 17). This effect was regioisomer-dependent, since replacement by a 3-THP had no impact on clearance (Figure 3A, row 19). Further examples are, however, required to confirm this difference in behavior. On the other hand, the use of cyclohexyl as a phenyl bioisostere had a small but detrimental effect on metabolic stability (Figure 3A, row 12).

The *para*-substituted benzene bioisosteres that could be assessed showed no significant impact on metabolic stability (Figure 3B, rows 5, 7, 10). Nonetheless, the number of examples reported is limited and this conclusion is thus preliminary. Conversely, the replacement of a *meta*-substituted benzene with a 1,3-disubstituted cyclohexane led to a decrease in clearance (Figure 3C, row 4) that is supportive of its incorporation when the objective is to increase the metabolic stability of lead compounds.

The next sections highlight recent, as well as particularly notable applications of benzene bioisosteres in drug design. These specific examples complement the general results of the data-mining analysis and showcase the utility of benzene bioisosteres in multiparameter optimization during drug optimization.

### Replacement of Terminal Benzene Rings

Among the scaffolds investigated, there were 23 different replacements for terminal benzene rings with five or more examples in ChEMBL (Figure 3A). Minimal effects on bioactivity were observed when using small carbocycles such as cyclopropane, cyclopentane, cyclohexane, as well as BCP as phenyl bioisosteres.

An example of the use of a cyclohexane as a phenyl bioisostere is demonstrated by Kitamura et al. in their development of noncovalent SARS-CoV-2 M^pro^ inhibitors.^55^ Their design was informed by the X-ray cocrystal structure of the protease bound to ML188 (**30**), which supported the addition of lipophilic groups to fill the S2 pocket (Figure 5). Indeed, the addition of either a phenyl or cyclohexyl group led to similar improvements in potency, showing the bioisosteric nature of these substituents. Nonetheless, the cyclohexane-based inhibitor **32** was cytotoxic and thus **31** was selected for further development. The proposed binding of the modified inhibitors was later confirmed through X-ray crystallography of compound **30** bound to SARS-CoV-2 M^pro^ (PDB: 3 V3M), which showed the phenyl group partaking in favorable hydrophobic interactions.

**Figure 5:**
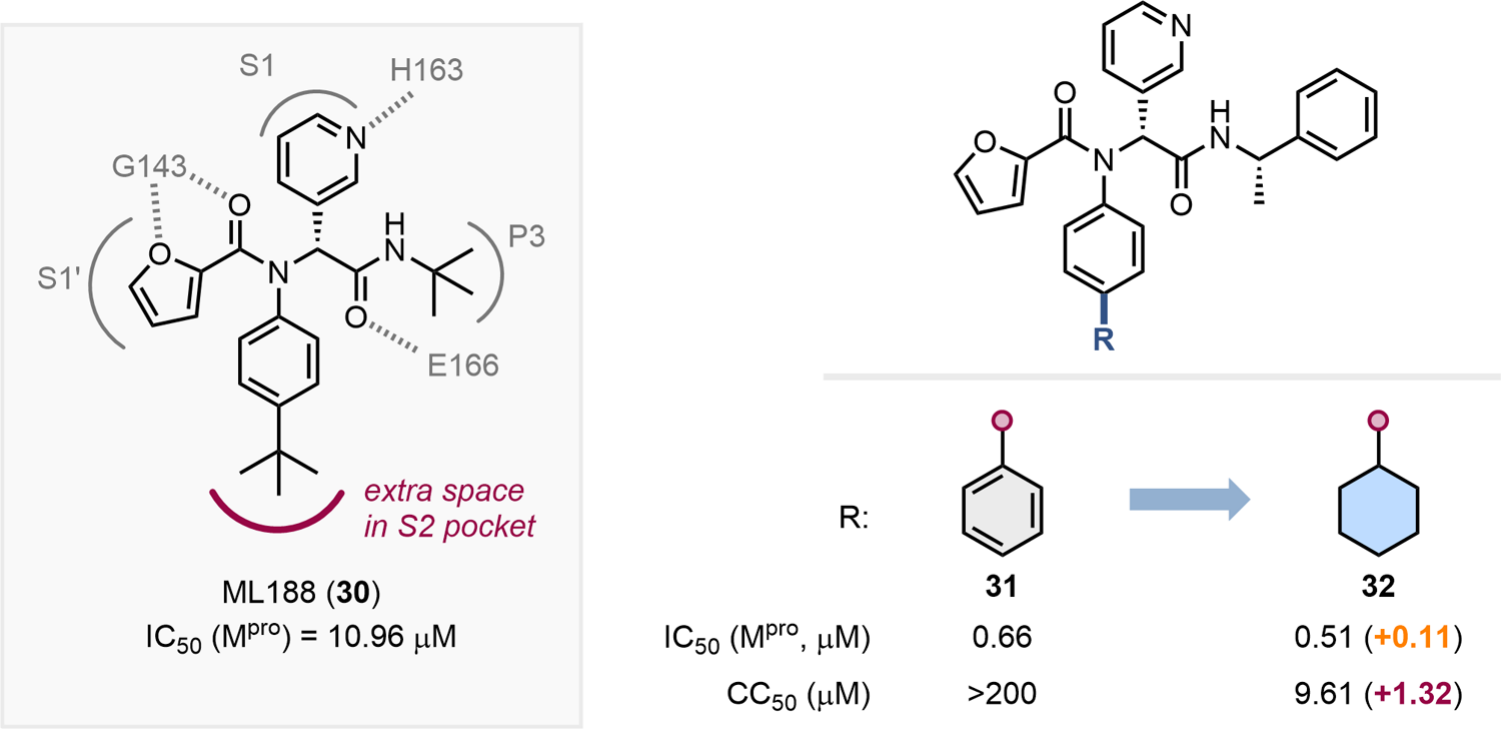
Schematic representation of the interactions between ML188 (**30**) and M^pro^ and impact on M^pro^ inhibition and cytotoxicity of the replacement of a phenyl substituent by a cyclohexyl ring.^55^

The effect of the replacement of a phenyl by a cyclohexyl ring on bioactivity is, nonetheless, context dependent. This is illustrated in Figure 6, which depicts the impact this substitution has on bioactivity across common target families (kinases, proteases, and family A GPCRs). The data show how proteases tend to tolerate this kind of substitution, as exemplified by SARS-CoV-2 M^pro^ cysteine protease inhibitors depicted in Figure 5, while in protein kinases this replacement has a significantly deleterious impact on bioactivity.

**Figure 6:**
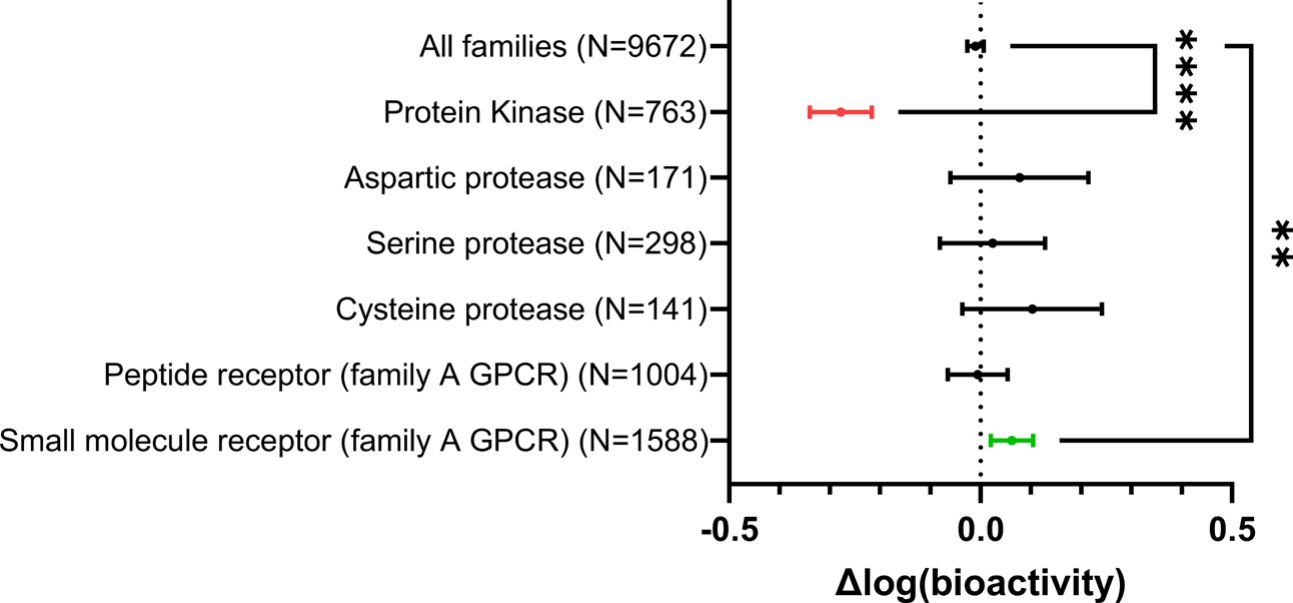
Impact of the substitution of a phenyl ring with a cyclohexane across target families. Data are presented as mean ± 95% confidence interval. Statistical significance was determined using one-way ANOVA followed by Dunnett’s multiple comparisons test. Significance is indicated as follows: *p* < 0.05 (*), *p* < 0.01 (**), *p* < 0.001 (***), *p* < 0.0001 (****).

The common negative impact of cyclohexane as a phenyl bioisostere in the context of protein kinase inhibition was exemplified by Pollard and colleagues at Vertex during their development of ataxia telangiectasia mutated and Rad3 related (ATR) protein kinase inhibitors (Figure 7).^56^ During their lead-optimization efforts, the authors sought to replace the anilide moiety in **33** with a bioisostere in order to avoid the formation of potentially toxic anilines *in vivo*. The replacement of the anilide with a cyclohexyl amide (**34**), nonetheless, resulted in a significant reduction in potency. A clash with the gatekeeper Tyr2365 residue may subtend this difference in bioactivity.

**Figure 7:**
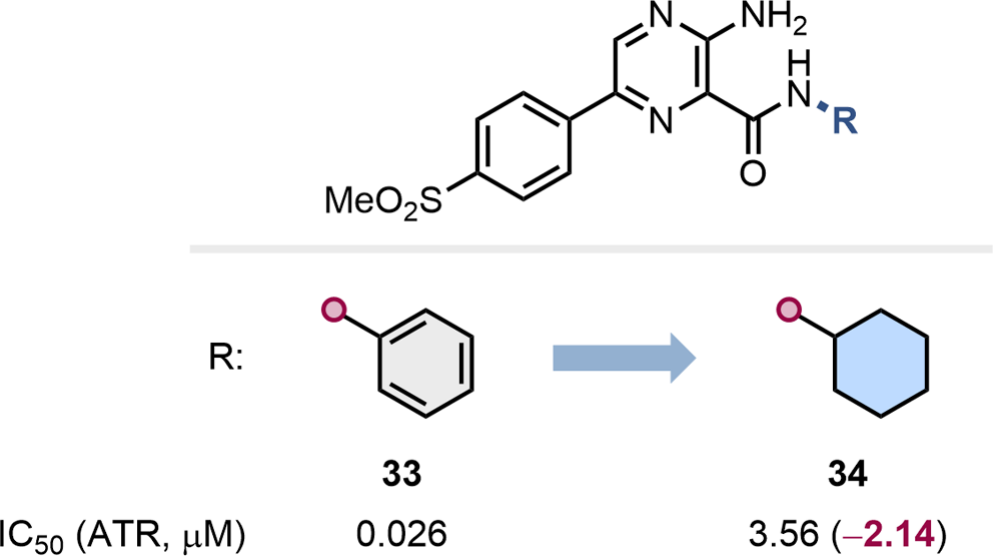
Effect of the replacement of a phenyl group with a cyclohexane on ATR inhibition.^56^

The replacement of a benzene ring by small aliphatic rings can lead to increased solubility, often due to reduced aromatic stacking in the solid state.^57^ This effect was most significant in our data-mining analysis for cyclopropane, as exemplified by Tear and co-workers in their optimization of small molecules for the treatment of human African trypanosomiasis (Figure 8).^58^ This transformation led to a small reduction in the pEC_50_ value but resulted in much improved solubility and a lower Log*D*. Opposing effects on metabolic stability were also observed that were species-dependent.

**Figure 8:**
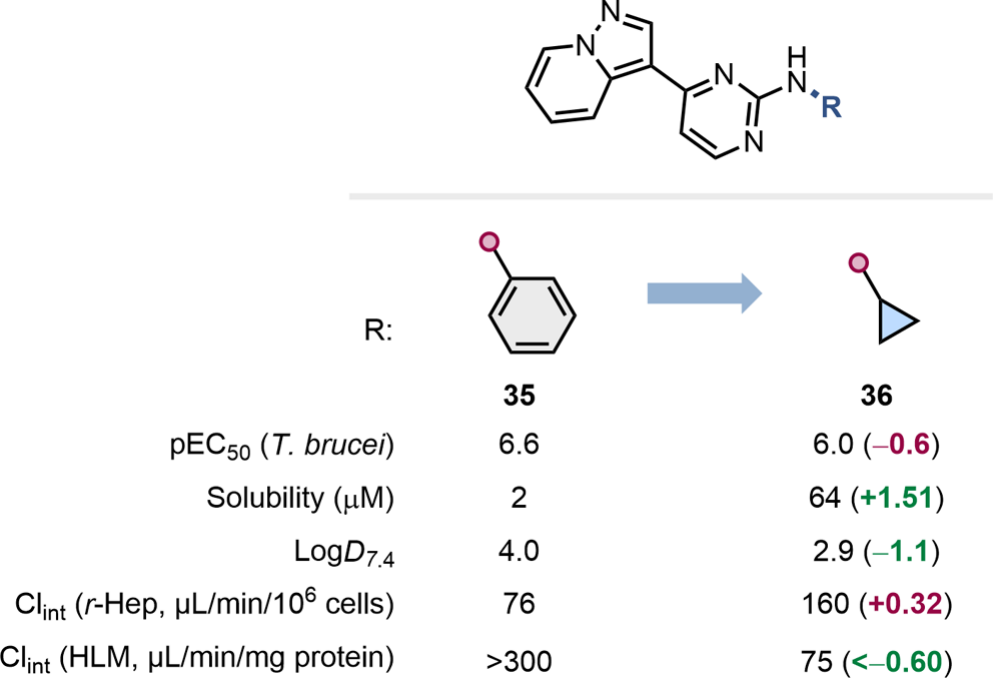
Impact of the replacement of a phenyl ring by a cyclopropyl group on T. brucei inhibitory potency, solubility, Log*D*, and clearance in rat hepatocytes and human microsomes.^58^

Partially saturated carbocycles may sometimes offer advantages as phenyl bioisosteres. This was showcased by Konteatis et al. in the discovery of AG-270, an oral methionine adenosyltransferase 2A (MAT2A) inhibitor for the treatment of tumors with homozygous methylthioadenosine phosphorylase (MTAP) deletion (Figure 9).^59^ Among the small rings explored, 1-substituted cyclohexene led to the biggest improvement in potency and was ultimately an element of the clinical candidate AG-270 (**42**).

**Figure 9:**
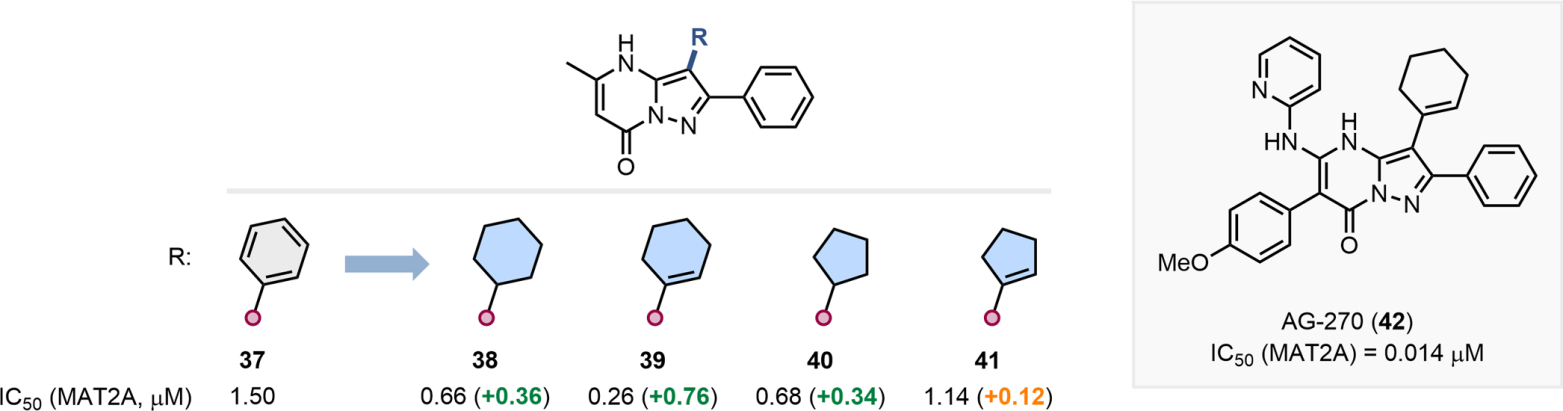
Impact of the replacement of a monosubstituted benzene with saturated and partially saturated carbocycles on MAT2A inhibition. Structure of the clinical candidate AG-270 (**42**).^59^

Bicyclic scaffolds such as BCPs, BCHs, BCHeps, and their heteroatom-containing analogues have recently experienced a resurgence in the literature, driven by the increase in methodologies for their synthesis and functionalization.^22^ So far, examples of their incorporation into bioactive molecules are scarce and thus their role as benzene bioisosteres remains tentative. Roecker and colleagues at Merck reported an example of the use of BCP as phenyl bioisostere as part of their development of Na_V_1.7 inhibitors (Figure 10).^60^ To improve the ADME properties of lead inhibitor **43**, the authors prepared a number of analogues **44**–**46** incorporating small alkyl motifs. Replacement of the phenyl moiety with cyclobutane (**44**) and cyclopropane (**45**) was detrimental to bioactivity, while incorporation of BCP (**46**) resulted in increased inhibitory potency toward Na_v_1.7. This replacement led to similar membrane permeability and reduced clearance when compared to the prototype **43**. Further development, informed by this series of small aliphatic replacements, led to the identification of **47** as an inhibitor with similar inhibitory potency to **43** and **46** but with more balanced properties that showed efficacy in rodent models of pain.

**Figure 10:**
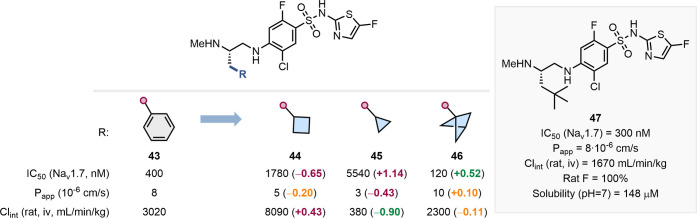
Impact of the switch from a phenyl to cyclobutane, cyclopropane, and BCP rings on the inhibition of Na_v_1.7, membrane permeability and clearance. Structure of the optimized compound **47**.^60^

Larger, hydrophobic groups have also been successfully employed as phenyl bioisosteres, as demonstrated by Roy et al. in their development of BCL-X_L_ and BCL-2 inhibitors (Figure 11).^61^ Their crystallography-guided approach led to the discovery of adamantyl-containing compound **49**, one of the most potent binders for this challenging PPI. The substitution of a phenyl group with adamantyl was designed to enhance hydrophobic interactions within a cryptic P5 pocket (highlighted in green in Figure 11), leading to improved potency toward BCL-X_L_ and MCL-1. Compounds **48** and **49** exhibited similar selectivity profiles against the BCL-2 family proteins, showing low binding to BCL-W or MCL-1, whereas the adamantyl-containing **49** demonstrated increased binding affinity for A1. The proposed binding mode of **49** to BCL-X_L_ was subsequently confirmed through X-ray cocrystallography (PDB: 6UVG) using the using the analogous but truncated inhibitor **50** (Figure 11).

**Figure 11:**
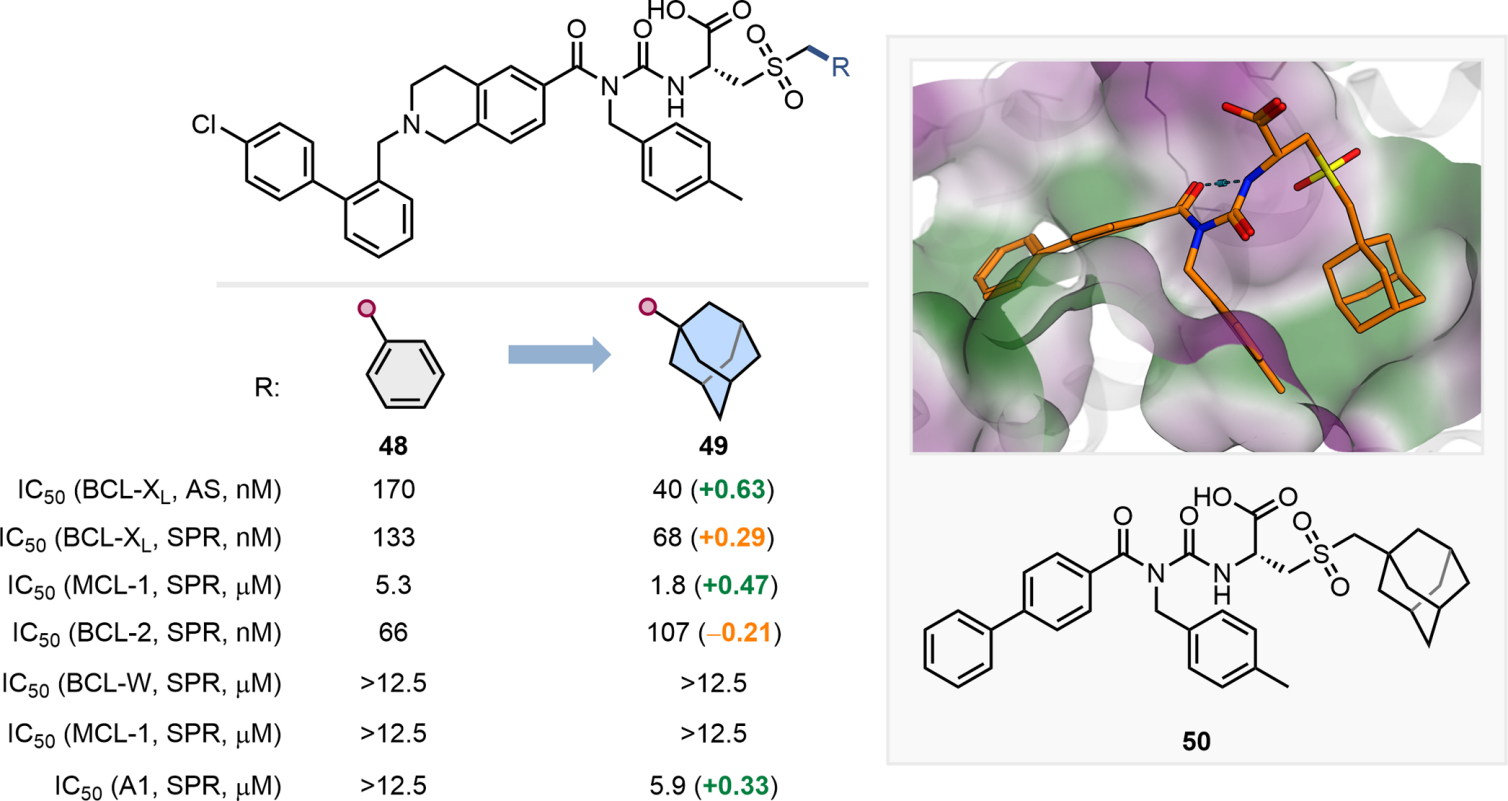
Effect of phenyl-to-adamantyl substitution on the inhibition of BCL-X_L_ and related BCL-2 family proteins. X-ray cocrystal structure of **50** (an analogue of **49**) bound to BCL-X_L_ shown in orange (PDB: 6UVG). Hydrophobic regions are highlighted in green and hydrophilic regions in purple.^61^

Todd and colleagues conducted a particularly elegant study exploring larger phenyl bioisosteres in a series of antimalarial agents incorporating carboranes, scaffolds that have found seldom use by medicinal chemists (Figure 12).^62^ In their effort to enhance the solubility and metabolic stability of a triazolopyrazine series, they replaced the phenyl ring with cubane, adamantane, and various *closo*-carboranes. Cubane substitution (**53**) preserved antimalarial activity whereas adamantane (**52**) was less well tolerated. Remarkably, incorporation of the rarely used 1,2- and 1,7-*closo*-carborane in **54** and **55**, respectively, led to significantly enhanced activity, while the 1,12-isomer (**56**) resulted in a reduction in inhibitory potency. The authors hypothesized that compound potency inversely correlated with the hydrophobicity of the carborane isomers. However, despite the promising bioactivity data, analogues **54** and **55** did not produce the desired improvements in solubility and metabolic stability.

**Figure 12:**
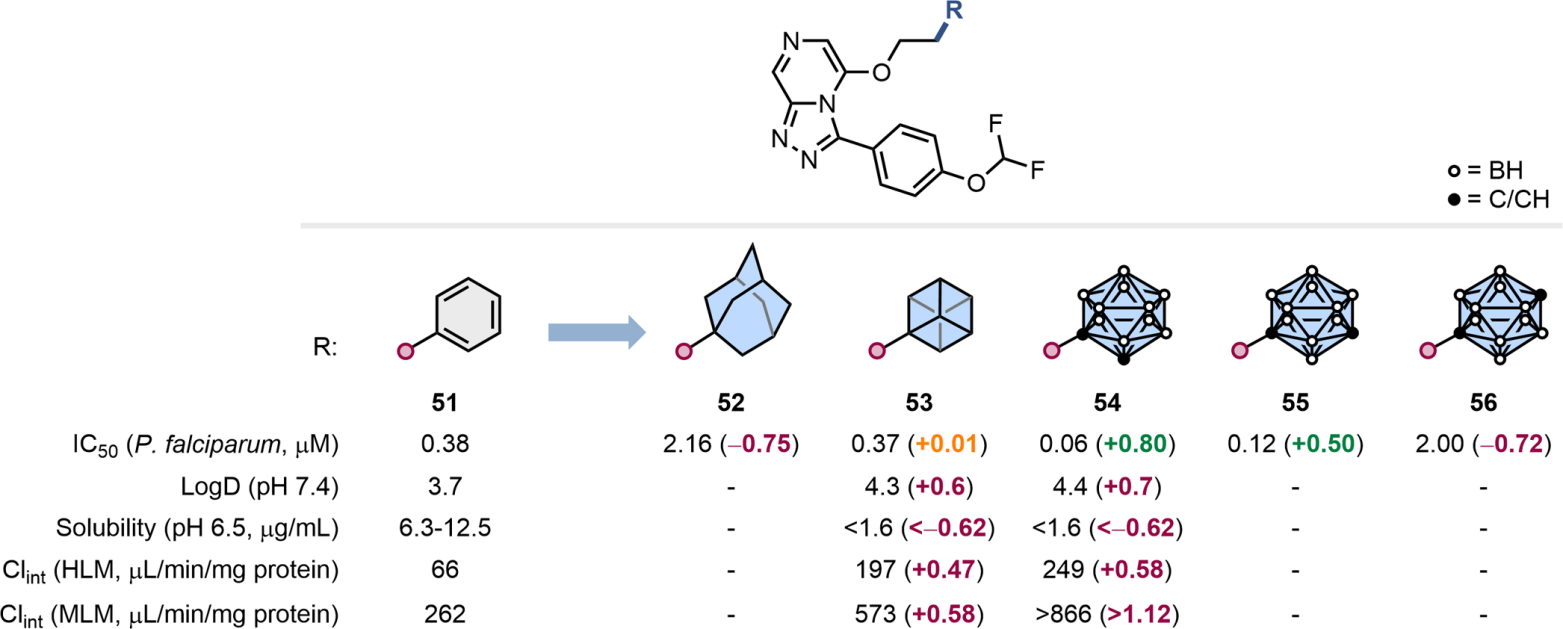
Effect of the replacement of a phenyl ring by adamantane, cubane, and carboranes on antimalarial activity, lipophilicity, solubility, and metabolic stability.^62^

The replacement of phenyl groups with heteroatom-containing ring systems such as THP, dioxanes, and piperazines, is less precedented and on average leads to reduced bioactivity. Thomas and co-workers replaced the phenyl ring in pyrazolopyrimidine **57** with a 4-substituted THP (**58**) as part of their development of treatments for visceral leishmaniasis (Figure 13).^63^ In this instance, the change resulted in a relatively small reduction in inhibitory potency, but was concomitant with a dramatic increase in solubility and much reduced mouse liver microsomal clearance.

**Figure 13:**
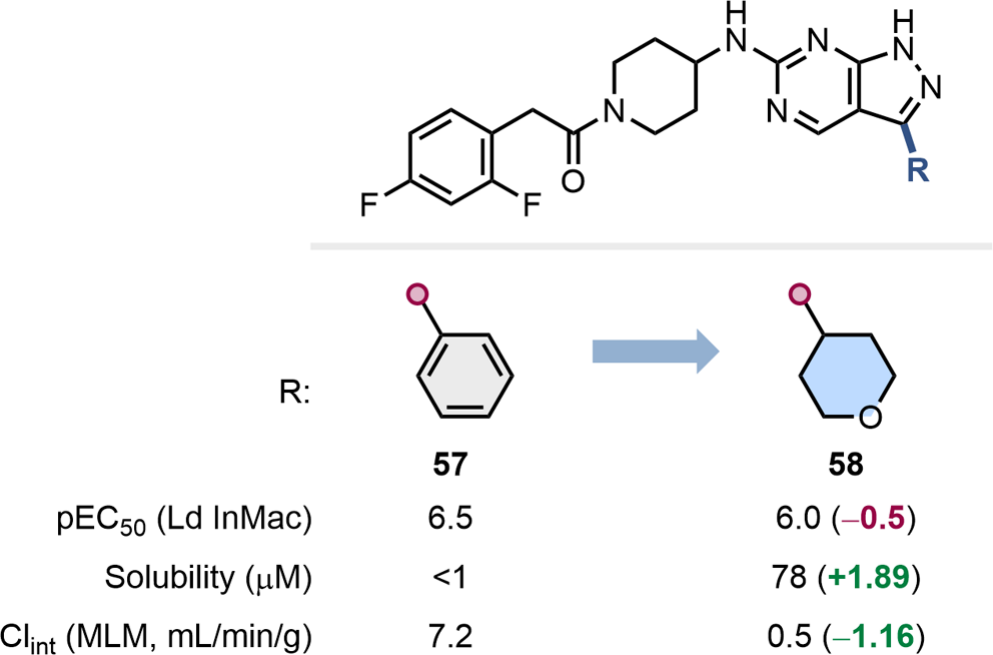
Impact of the substitution of a phenyl group by 4-THP as part of the development of treatments against visceral leishmaniasis.^63^

### Replacement of Para-Substituted Benzene Rings

Based on our data-mining analysis, the replacements for a *para*-substituted benzene with the smallest impact on bioactivity were 1,3-substituted cyclopentane, 1,4-substituted cyclohexane, 1,4-substituted cyclohexene, and 1,3-substituted cyclobutane (Figure 3). Both 1,4-substituted cyclohexane and a disubstituted BCP led to significant increases in solubility, while none of the bioisosteres investigated had a significant impact on metabolic stability.

Huang et al. employed 1,4-substituted cyclohexane as a *para*-substituted benzene bioisostere in their development of tankyrase (TNKS) inhibitors (Figure 14).^64^ In line with our analysis, the *trans* isomer **60** outperformed the *cis* isomer **61** across all assays. The X-ray cocrystal structures of **59** and **60** complexed with TNKS1 showed good overlap between the *para*- substituted benzene in **59** and the *trans*-1,4-cyclohexane ring in **60**, supporting the role of this scaffold as bioisosteric.

**Figure 14:**
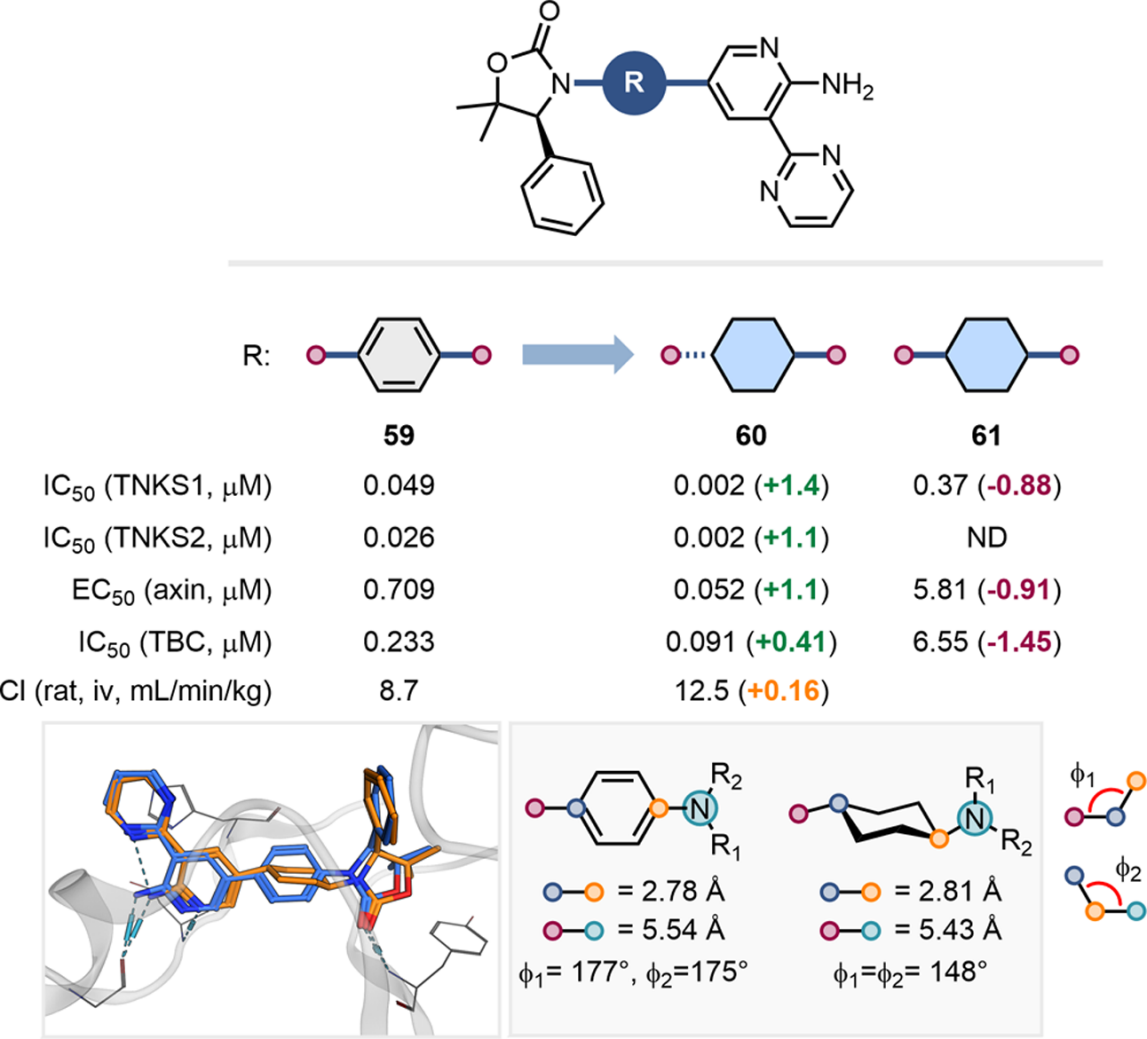
Impact on TNKS inhibitory activity and clearance of the replacement of a para-substituted benzene with cis- and trans-1,4-substituted cyclohexane. X-ray cocrystal structures of **59** (blue, PDB: 4N4T) and **60** (orange, PDB:4N4 V) with TNKS1. Exit vector analysis of para- substituted benzene and trans-1,4-substituted cyclohexane (measurements extracted from the cocrystal structures).^64^

Pu and colleagues demonstrated the utility of a disubstituted BCP as a *para-*substituted benzene bioisostere in their development of indoleamine-2,3-dioxygenase 1 (IDO1) inhibitors (Figure 15).^65^ In their study, replacing the *para*-substituted benzene in **62** with a BCP reduced the ALog*P* value by over one logarithmic unit and resulted in a slight reduction in potency. X-ray cocrystallographic analysis of **62** and **63** bound to IDO1 highlighted the similar angles of the exit vectors of these scaffolds (*ca*. 180°), and the shorter distance between them in **63** (2.84 vs 1.93 Å). The shorter distance between hydrogen-bond acceptors may preclude their effective interaction with both water molecules in IDO1, thus providing a plausible explanation for the lower inhibitory potency of BCP-containing analogue **63**. Further optimization of the BCP-containing series led to the discovery of the potent, orally available BCP analogue **64**, which was equipotent to the initial hit **62** but with significantly lower clearance. The authors attributed this improvement to slower amide hydrolysis as a result of the incorporation of the BCP core.

**Figure 15:**
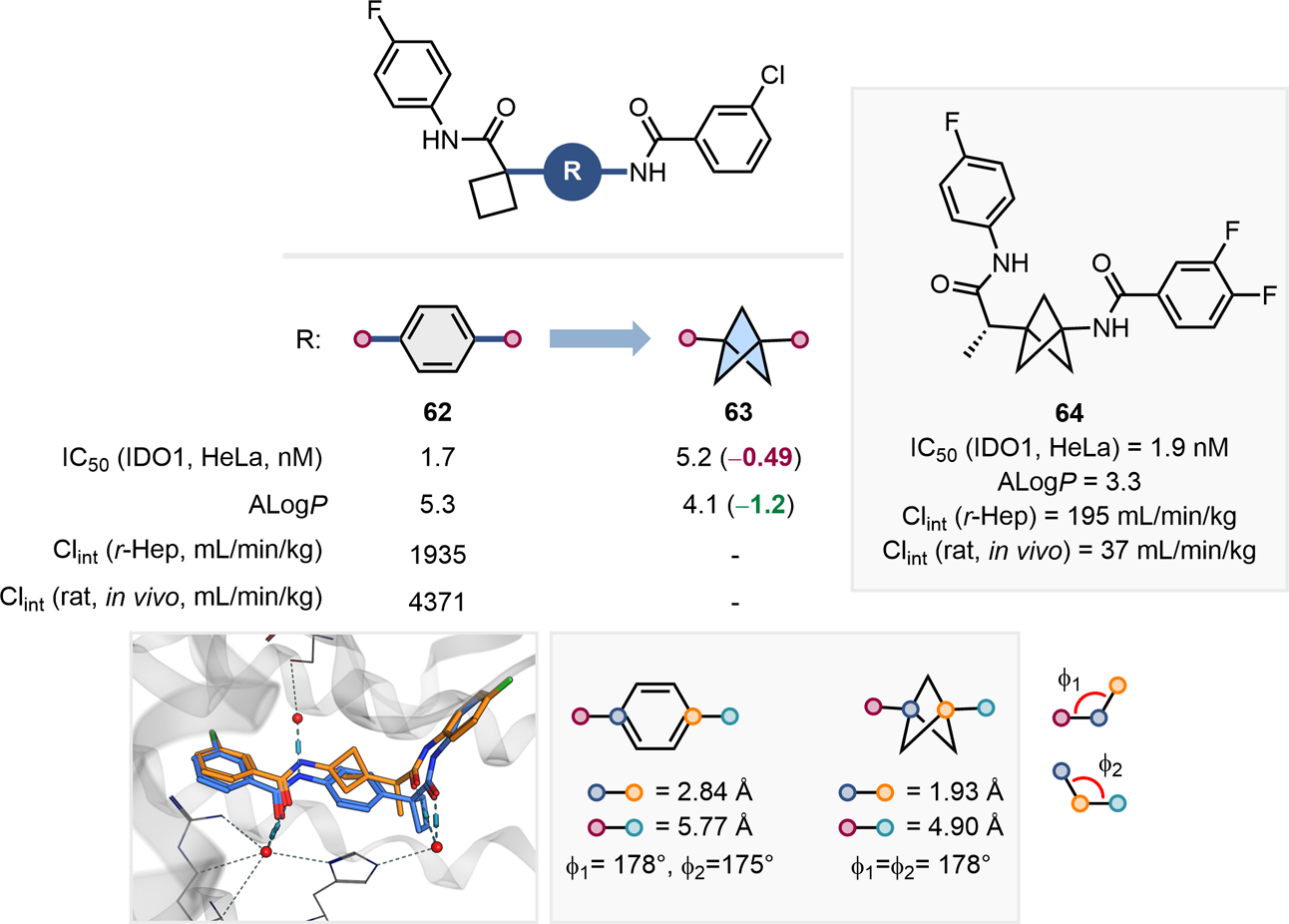
Effect of the replacement of a para-substituted benzene ring with a disubstituted BCP on IDO1 inhibitory activity, ALog*P*, and Cl_int_ and further development toward a candidate molecule **64**. X-ray cocrystal structures of **62** (blue, PDB: 6 V52) and **63** (orange, PDB: 6WJY) with IDO1. Exit vector analysis of para-substituted benzene and 1,3-BCP (measurements extracted from the cocrystal structures).^65^

Aguilar and co-workers demonstrated the utility of a [2.2.2]­BCO as a bulkier *para*-substituted benzene bioisostere in the development of murine double minute 2 (MDM2) inhibitors (Figure 16).^66^ The authors’ extensive SAR study included MMPs in which the benzene ring of **65** was replaced with various disubstituted aliphatic scaffolds. Replacement with *trans*-1,4-cyclohexane yielded equipotent compound **66** while the *cis*-isomer **67**, consistent with our analysis, exhibited reduced bioactivity across assays. BCP-containing analogue **68** showed comparable *in vitro* binding affinity but was less potent in the cell-based assay (SJSA-1 xenograft model). Finally, the [2.2.2]­BCO-containing compound **69** maintained similar MDM2 affinity and growth inhibition to benzoic acid **65**, and had much reduced clearance in liver microsomal preparations. However, despite its promising pharmacokinetic profile, compound **69** lacked *in vivo* efficacy, with no observed tumor regression – an outcome that the authors attributed to poor tissue penetration. *N*-Alkylation of the pyrrolidine ring in compound **69** circumvented this tissue penetration issue and led to **70**, which showed 100% tumor regression in rat *in vivo* studies. Compound **70** has since advanced into phase II clinical trials for the treatment of relapsed/refractory T-cell prolymphocytic leukemia (R/R T-PLL) and non-Hodgkin’s lymphoma (NHL).^67^

**Figure 16:**
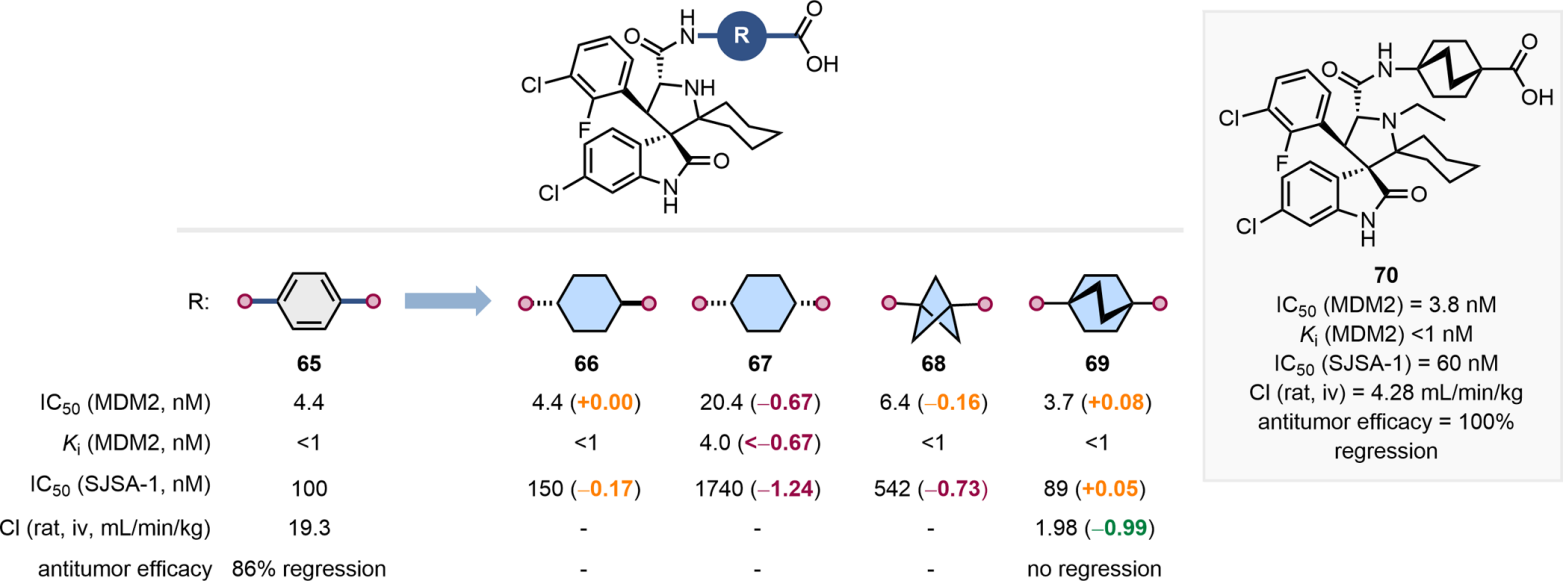
Impact of replacing a para-substituted benzene ring with several disubstituted aliphatic scaffolds on MDM2 affinity, cellular activity, pharmacokinetic parameters, and in vivo efficacy. Structure of the resulting clinical candidate **70**.^66^

Partially saturated carbocycles can also be employed as *para*-substituted benzene bioisosteres, as showcased by Swidorski and co-workers in their development of broad-spectrum HIV-1 maturation inhibitors (Figure 17).^68,69^ Aromatic ring-containing triterpenoid GSK3532795 (**71**) was a candidate that advanced into phase IIb clinical trials; however, development was halted due to gastrointestinal intolerability and treatment-emergent resistance. The replacement of the *para*-substituted benzene ring with nonaromatic motifs was explored as a strategy to ameliorate these issues. Indeed, incorporation of 1,4-substituted cyclohexene (**72**) or 2,6-substituted spiro[3.3]­hept-1-ene (**73**) resulted in improved potency across the HIV-1 mutant strains tested. Further development of cyclohexene **72** led to the discovery of GSK3640254 (**74**), a second clinical candidate to reach clinical trials, which did not show treatment-emergent resistance in early clinical studies. In 2024, the authors disclosed a third clinical candidate incorporating a cyclohexene ring with further improved antiviral properties and the potential for once-weekly dosing.^70^

**Figure 17:**
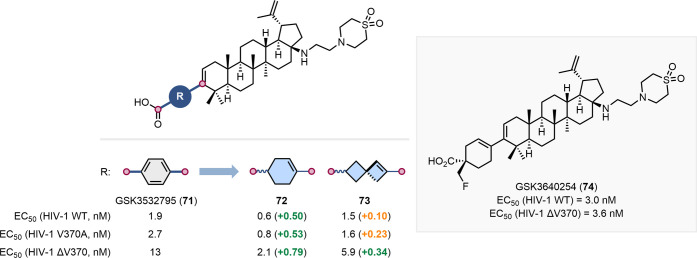
Effect of the replacement of a para-substituted benzene with a 1,4-substituted cyclohexene and 2,6-substituted spiro[3.3]­hept-1-ene in the development of broad spectrum HIV-1 maturation inhibitors. Structure of the clinical candidate GSK3640254 (**74**).^68,69^

1,4-Piperazine generally leads to a loss in bioactivity when employed as a *para*-substituted benzene bioisostere, as evidenced by our MMP analysis (Figure 3). This is often due to its basicity, which results in the ammonium ion being the predominant species in solution at physiological pH. Substitution of piperazines with electron withdrawing groups can, however, lower their p*K*_a_ values and allow their productive application as *para*-substituted benzene bioisosteres. Klug and co-workers’ development of small-molecule treatments for human African trypanosomiasis, which is caused by the protozoan parasite *Trypanosoma brucei* (*T. brucei*), exemplifies this approach (Figure 18). The authors replaced the *para*-substituted benzene ring in azaindole **75** with the 1,4-piperazine in **76** which, due to the methanesulfonyl substituent, is associated with attenuated basicity. This replacement resulted in a small drop in bioactivity, but was accompanied by an increase in solubility.^71^

**Figure 18:**
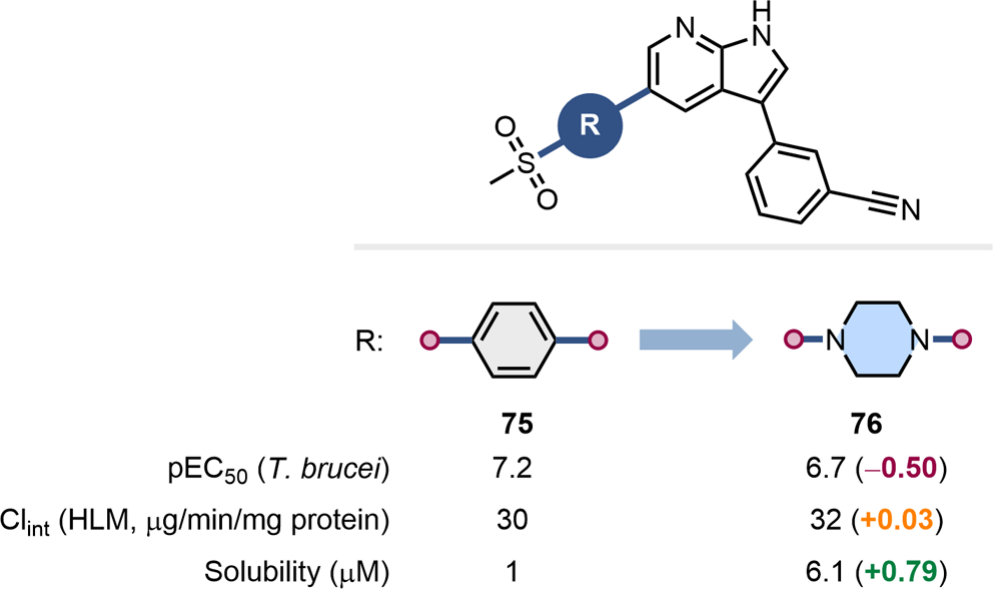
Impact of the substitution of a para-substituted benzene with a 1,4-piperazine heterocycle in the context on inhibitory activity toward *T. brucei*, clearance, and solubility.^71^

### Replacement of Meta-Substituted Benzene Rings

Our analysis showed that carbocycles such as 1,3-substituted cyclobutane, 1,3-substituted cyclopentane, and 1,3-substituted cyclohexane had the smallest impact on bioactivity when used as *meta-*substituted benzene bioisosteres. Furthermore, 1,3-substituted cyclopentane led to a significant increase in solubility.

An elegant example of the utility of 1,3-cyclohexanes as *meta*-substituted benzene bioisosteres is their incorporation into SETD2 inhibitors, as demonstrated by Farrow and co-workers (Figure 19).^72^ The bis-aniline core in lead compound **77** led to poor pharmacokinetic properties and potential metabolism-derived toxicity, prompting the screening of alternative, saturated scaffolds to address these limitations. Substituting the problematic *meta-*substituted benzene ring with a 1,3-substituted cyclohexane yielded the clinical candidate EZM0414 (**78**), which retained similar potency in biochemical assays while demonstrating enhanced antiproliferative activity and an improved PK profile. The relative and absolute configuration of the stereocenters on the cyclohexane ring had a critical impact on bioactivity. X-ray cocrystal structures of **77**, **78**, and **80** bound to SETD2 revealed that the exit vectors of the *cis*-isomer, bearing the (1*R*,3*S*) configuration, closely mimicked those of the *meta*-substituted benzene ring, providing structural insight into its functional mimicry (Figure 19).

**Figure 19:**
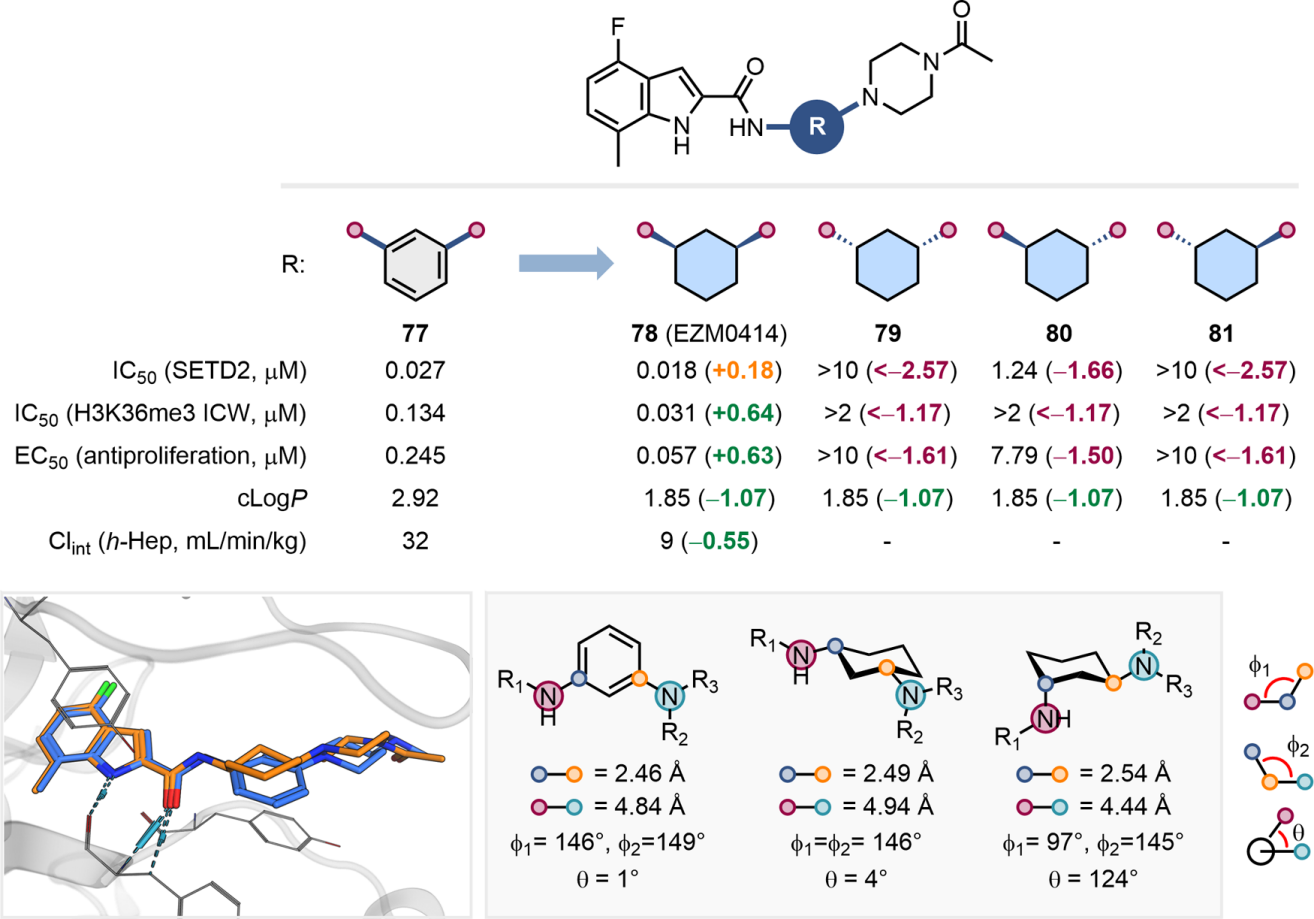
Effect on SETD2 inhibition, cLog*P*, and clearance of the replacement of a meta- substituted benzene with a 1,3-substituted cyclohexane. X-ray cocrystal structures of an analogue of **77** (blue, PDB: 7LZD) and EZM0414 (**78**) (orange, PDB: 7TY2) with SETD2. Exit vector analysis of meta-substituted benzene, cis- and trans-1,3-substituted cyclohexane (measurements extracted from the cocrystal structures (PDB: 7LZD, 7TY2, 7TY3)).^72^

Munck af Rosenschö and colleagues at AstraZeneca showcased the bioisosteric nature of cyclopropanes in their development of leukotriene C4 synthase (LTC4S) inhibitors (Figure 20).^73^ The motivation behind this replacement strategy was to improve the properties of **82** to allow oral administration. Indeed, substitution of the *meta*-substituted benzene ring with a *trans*-cyclopropane to give carboxylic acid **83** resulted in the maintenance of inhibitory activity across both the enzyme and cell-based assays, along with reduced Log*D* and lower hepatic clearance. Further elaboration of **83** resulted in the invention of the clinical candidate **84**, which was progressed into phase I clinical studies.^74^

**Figure 20:**
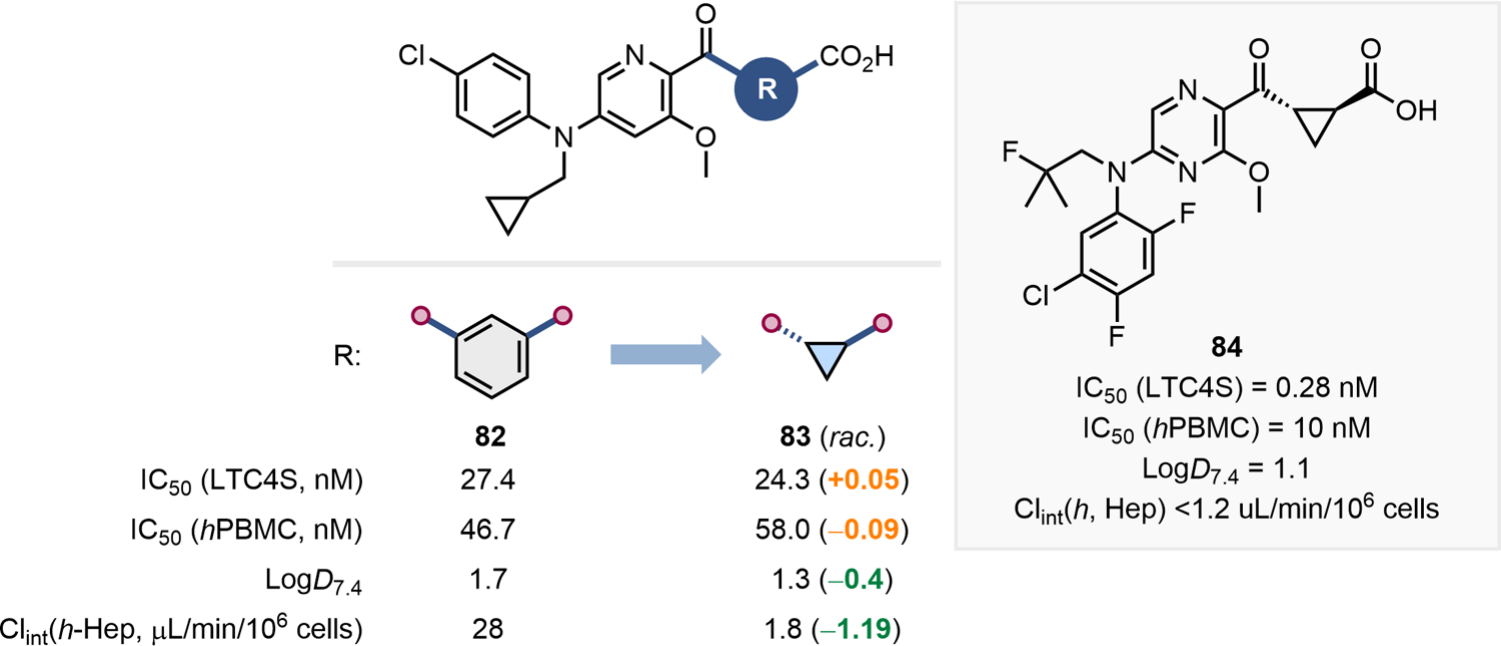
Impact of the switch from a meta-substituted a benzene to a cyclopropane on LTC4S inhibition, Log*D*, and hepatic clearance. Structure of the clinical candidate **84** resulting from optimization of **83**.^73^

Scientists at Bristol Myers-Squibb reported the effects of the replacement of a *meta-*substituted benzene with a cyclopentane and a [2.2.1]­BCH as part of their development of interleukin-1 receptor-associated kinase 4 (IRAK4) inhibitors (Figure 21).^75^ In this context, the three scaffolds led to similarly potent compounds in the biochemical assays, while in the cell-based assay the compound bearing a *trans*-1,2-cyclopentane (**87**) was less potent than **85**, **86**, and **88**. The four diastereoisomers of the 1,3-substituted cyclopentane **86** were similarly active, with differences being more pronounced in the cell-based assay (data not shown).^75^

**Figure 21:**
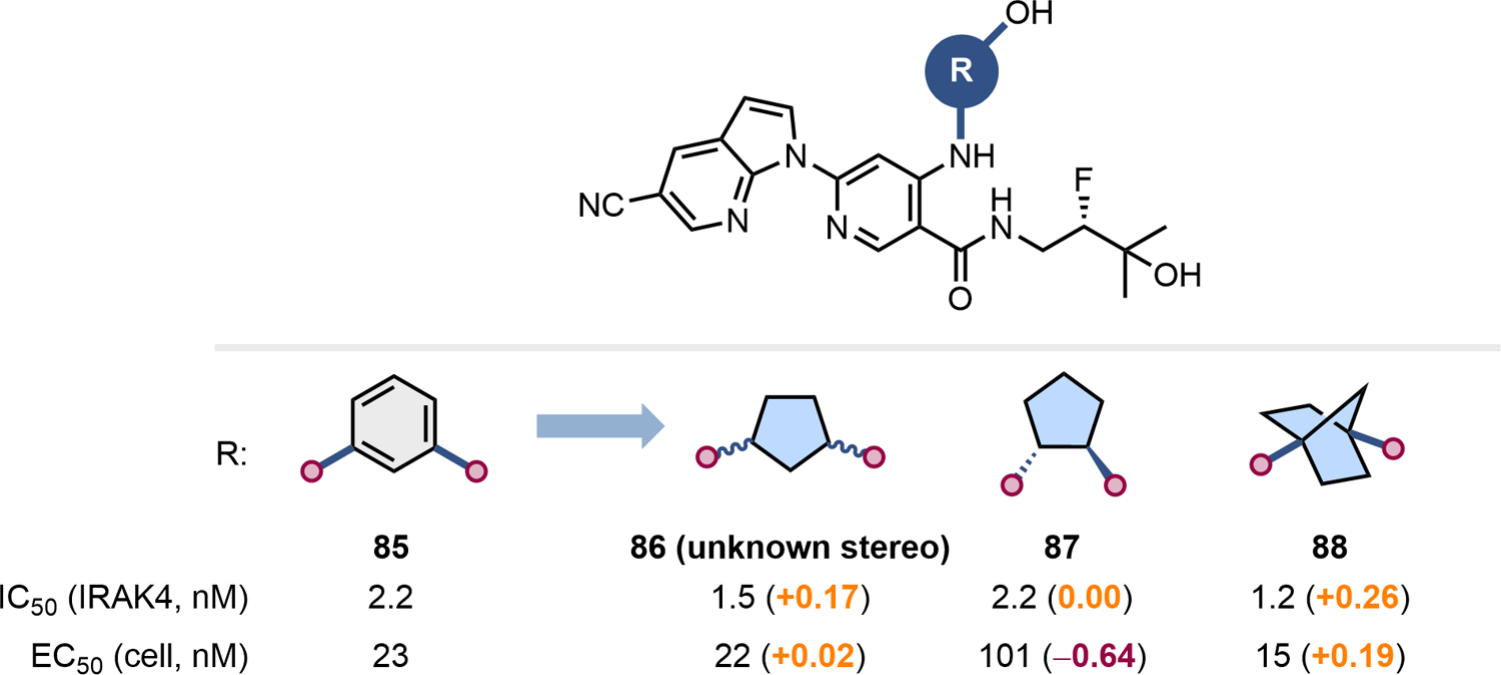
Impact of the replacement of a meta-substituted benzene with cyclopentane and [2.2.1]­BCH on IRAK4 inhibition.^75^

Although less precedented, likely due to the scarcity of methods for the synthesis of disubstituted scaffolds, caged ring systems such as BCPs, BCHs, BCHeps and cubanes, have potential as *meta*-substituted benzene bioisosteres. This was exemplified by Caldwell et al. in the development of covalent Bruton’s tyrosine kinase (BTK) inhibitors (Figure 22).^76^ To improve the aqueous solubility and metabolic stability of acrylamide **89**, the authors explored the replacement of the *meta*-substituted benzene ring with alternative alkyl linkers. This was supported by SAR and X-ray cocrystal structure data. Incorporation of a [2.1.1]­BCH linker (**90**) resulted in increased solubility and membrane permeability, and lower hepatic clearance, concomitant with a small reduction in inhibitory potency in both the enzyme and cell-based assays. Further exploration of the linkers led to the discovery of evobrutinib (**91**), a BTK inhibitor with higher solubility and membrane permeability that was progressed to clinical trials.

**Figure 22:**
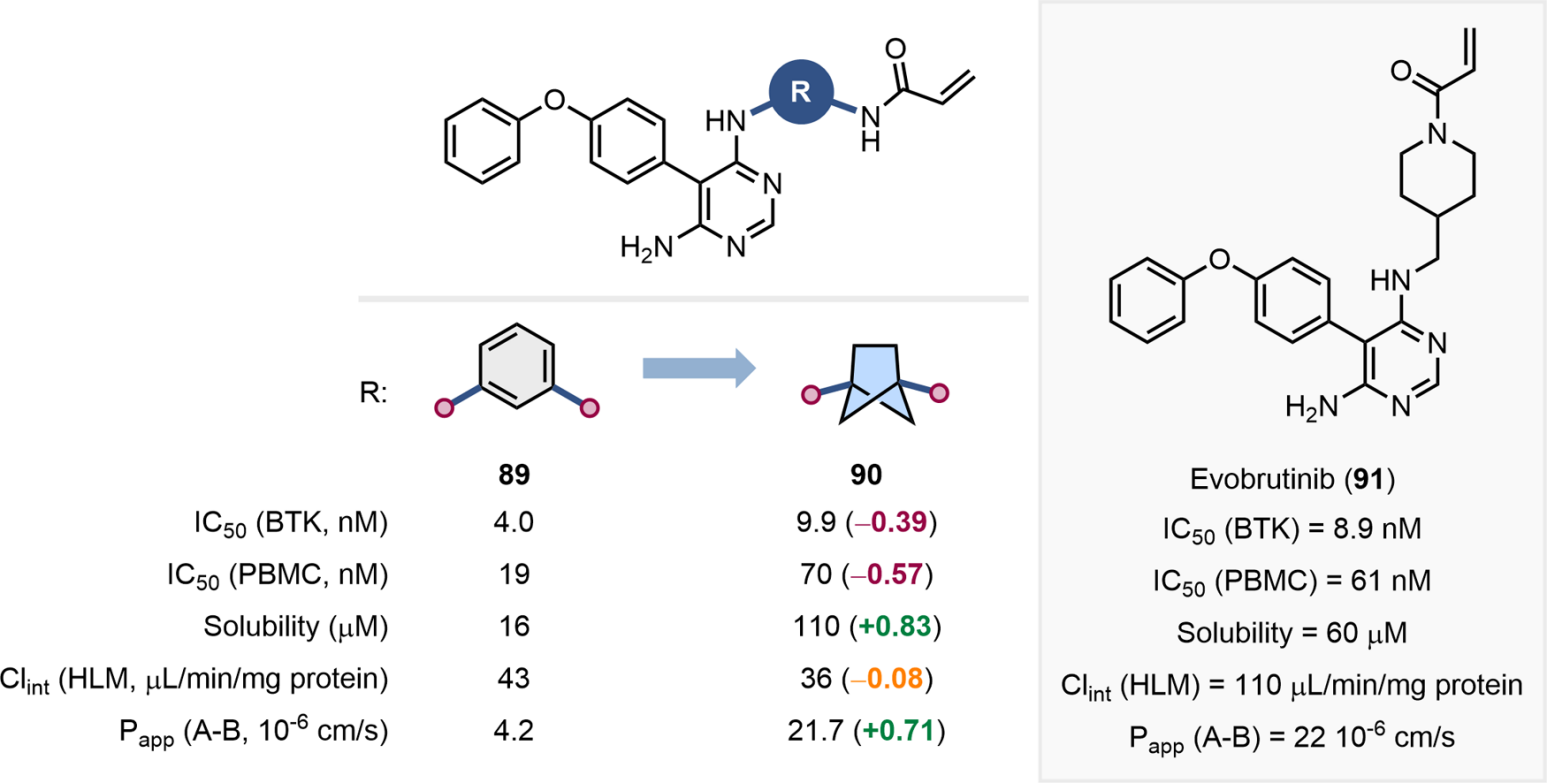
Effect of replacing a meta-substituted benzene ring with a [2.1.1]­BCH linker on BTK inhibition, solubility, hepatic clearance, and membrane permeability. Structure of clinical candidate evobrutinib (**91**).^76^

### Replacement of *ortho*-Substituted Benzene Rings

There are limited data available in ChEMBL on the application of *ortho*-substituted benzene bioisosteres. Our workflow showed that 1,2-substituted cyclohexane and 1,2-substituted cyclohexene are the replacements that lead to smallest changes in bioactivity, although these effects were context dependent. The incorporation of a 1,2-substituted cyclohexane was associated with an increase in solubility.

Kuduk and co-workers aimed to replace the *ortho-*substituted benzene ring in the bradykinin B_1_ antagonist **92** with an alternative, nonaromatic scaffold to improve the physicochemical properties of their compounds (Figure 23).^77^ Toward this aim, compounds **93**–**97** were prepared. The replacement of the *ortho*-substituted benzene with a 1,2-substituted cyclohexene (**93**) or a bicyclo[2.2.1]­heptane (**97**) led to a significant reduction in inhibitory potency. On the other hand, the 4,5-substituted cyclohexenes **94**–**95** and the *trans*-1,2-substituted cyclohexane **96** showed improved binding affinity for the bradykinin B1 receptor, although this was concomitant with increased clearance *in vivo*. The authors speculated that the nonaromatic nature of the methyl ester in **94** and **96** resulted in faster hydrolysis, responsible for their lower metabolic stability compared to the *ortho*-substituted benzene **92**.

**Figure 23:**
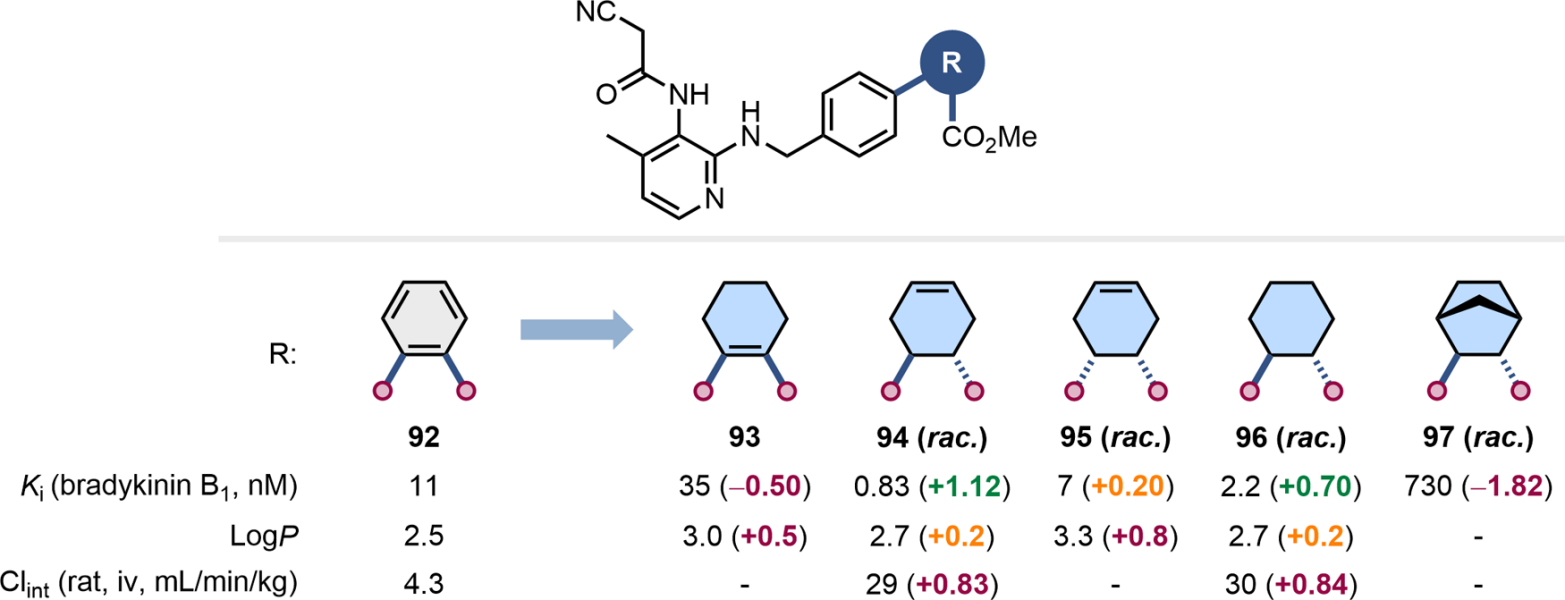
Impact of the replacement of an ortho-substituted benzene with alternative nonaromatic scaffolds on antagonism of bradykinin B1 receptors, Log*P*, and metabolic stability.^77^

Smaller rings, such as cyclopropanes and cyclopentanes, can also be used to replace *ortho*-substituted benzenes, as demonstrated by Saleeb et al. in their development of inhibitors of *Pseudomonas aeruginosa* exoenzyme S ADP-ribosyltransferase activity (Figure 24).^78^ In this example, the relative configuration of the cyclopropane ring had a significant impact on bioactivity, with the *trans* isomer **99** outperforming the *cis*-isomer **100** by an order of magnitude.

**Figure 24:**
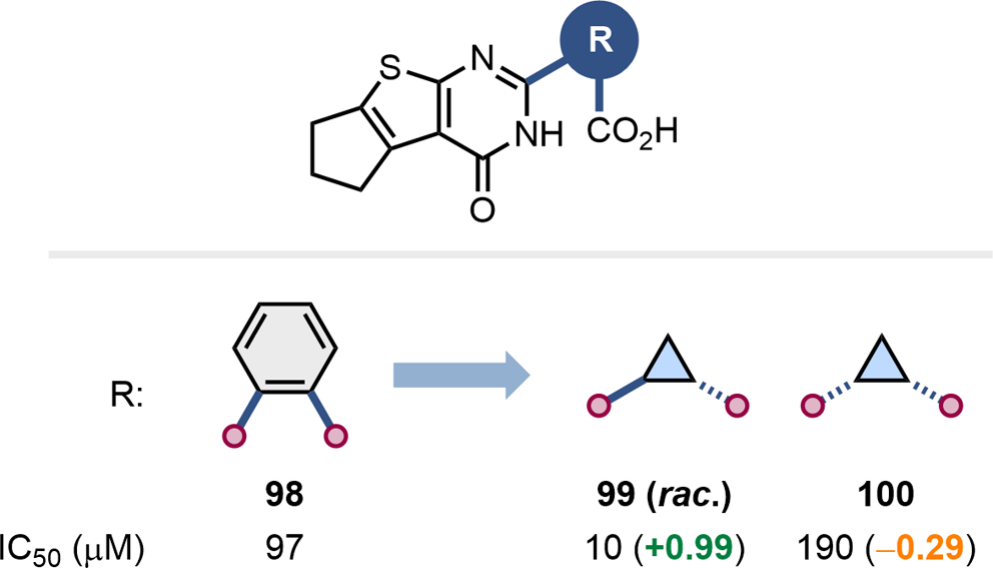
Substitution of an ortho-substituted benzene with cyclopropane in inhibitors of *Pseudomonas aeruginosa* exoenzyme S ADP-ribosyltransferase.^78^

## Summary and Outlook

In this perspective, we lay out a strategy to mapping the bioisostere landscape through a data-driven approach, exemplified by mapping benzene bioisosteres. This allowed us to evaluate the context-dependency of such bioisosteric substitutions while highlighting recent applications and identifying areas that would benefit from further research. This was achieved through the deployment of an open-access, data-mining workflow (BioSTAR).

The data-mining analysis performed enabled the ranking of 57 potential benzene bioisosteres based on their impact on bioactivity, solubility and clearance. We hope that these findings will assist practicing medicinal chemists in prioritizing potential bioisosteric replacements for benzene, and that the data-mining workflow employed will serve as a valuable, general tool for the quantitative evaluation of molecular replacements.

Examining the benzene bioisostere landscape revealed areas with limited available data, highlighting opportunities where additional research could significantly benefit the community. For example, bioactivity data for *meta*- and *ortho*-substituted benzene bioisosteres remains particularly scarce. The development of synthetic methodologies to access novel, and diversely functionalized carbocyclic scaffolds is therefore essential to advance this field and allow the evaluation of the impact of these replacements on bioactivity, solubility, and ADME properties.

The findings of this data-mining analysis are expected to evolve as more data becomes available. In line with this, the workflow developed only uses open-source software so others may reproduce and extend this analysis over time or apply it to different open access or even proprietary databases. Expanding this approach to include the extensive bioactivity and molecular property data from pharmaceutical companies holds great promise. A notable, relevant example is the work by Kramer et al., who conducted a MMP analysis to extract absorption, distribution, metabolism, elimination and toxicity (ADMET) knowledge from pooled data shared by AstraZeneca, Genentech, and Roche.^79^ This collaboration resulted in synergistic knowledge gains without compromising intellectual property, as full molecular structures were not disclosed. Applying a similar effort to bioactivity and bioisosterism could yield equally promising results, advancing the field while preserving proprietary information.

## Supplementary Material



## Notes

Extended results of the MMP analysis, analysis of the impact of stereochemistry on bioactivity, correlation analysis between compound properties and cLog*P*, and BioiSostere Analysis and Ranking (BioSTAR) workflow user guide (PDF)

## Abbreviations Used

ADME: absorption, distribution, metabolism and elimination
ADMET: absorption, distribution, metabolism, elimination and toxicity
ANOVA: analysis of variance
ATR: ataxia telangiectasia mutated and Rad3 related
BCH: bicyclo­[2.1.1]­hexane
BCHep: bicyclo­[3.1.1]­heptane
BCO: bicyclo­[2.2.2]­octane
BCP: bicyclo­[1.1.1]­pentane
BioSTAR: BioiSosTere Analysis and Ranking
BTK: Bruton’s tyrosine kinase
F+I: fragmentation and indexing algorithm
Fsp^3^: the fraction of carbon atoms in a molecule that are *sp* ^3^ hybridized
IDO: indoleamine-2,3-dioxygenase
IRAK: interleukin-1 receptor-associated kinase
LTC4S: leukotriene C4 synthase
MCS: maximum common substructure
MDM: murine double minute
MMP: matched molecular pair
MAT2A: methionine adenosyltransferase 2A
MTAP: methylthioadenosine phosphorylase
NHL: non-Hodgkin’s lymphoma
PDB: protein data bank
R/R T-PLL: relapsed/refractory T-cell prolymphocytic leukemia
*T. brucei*: *Trypanosoma brucei*
THP: tetrahydropyran
TNKS: tankyrase
tPSA: topological polar surface area
