# Supplementary material for: A Data-Driven Perspective on Bioisostere Evaluation: Mapping the Benzene Bioisostere Landscape with BioSTAR
Source: J Med Chem. 2025 Aug 5;68(16):16921–39. doi: 10.1021/acs.jmedchem.5c01641 (PMC12406194; doi:10.1021/acs.jmedchem.5c01641)
Supplement: Supplementary file 1 [file jm5c01641_si_001.pdf]

Supporting Information

A Data-Driven Perspective on Bioisostere Evaluation: Mapping the Benzene Bioisostere Landscape with BioSTAR

Pol Hernández-Lladó*†, Nicholas A. Meanwell§ & Angela J. Russell*†‡

† *Department of Chemistry, Chemistry Research Laboratory, University of Oxford, Mansfield Road, Oxford OX1 3TA, United Kingdom.*

‡ *Department of Pharmacology, University of Oxford, Mansfield Road, Oxford OX1 3QT, United Kingdom.*

§ *The Baruch S. Blumberg Institute, 3805 Old Easton Road, Doylestown, Pennsylvania 18902, United States; The School of Pharmacy, The University of Michigan, Ann Arbor, Michigan 48109, United States; The Ernest Mario School of Pharmacy, Rutgers University, Piscataway, New Jersey 08854, United States; NuArq MedChem Consulting LLC, Yardley, Pennsylvania 19067, United States.*

*****E-mail: pol.hernandezllado@chem.ox.ac.uk; angela.russell@chem.ox.ac.uk

Contents

1. Results of the MMP analysis S2
2. Analysis of the impact of stereochemistry on bioactivity S7
3. Correlation analysis between compound properties and cLogP S8
4. BioiSosTere Analysis and Ranking (BioSTAR) workflow user guide S9

1. Results of the MMP analysis

Table S1. Summary of the results obtained for bioactivity of mono-substituted benzene rings. Mean values are plotted with error bars representing 95% confidence intervals.[a] Mean of Δ(−log(bioactivity)); [b] A one-sample *t*-test was performed to assess whether the mean significantly differs from zero; [c] Number of protein target families as per ChEMBL classification (level 3); [d] Welch's ANOVA was used to test whether mean bioactivity changes differed significantly across target families, accounting for unequal variances.

Entry	Transformation	Mean difference (95% CI)	N	Mean[a]	SD	P value[b]	Target Families[c]	Context effects (P value)[d]
1	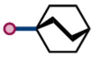		7	1.38	0.57	0.001	2	-
2	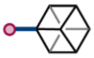		14	0.85	0.62	0.0002	4	-
3	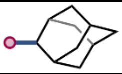		98	0.34	0.95	0.001	12	Y (0.010)
4	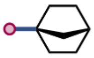		5	0.28	0.73	0.439	-	-
5	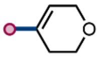		48	0.27	0.92	0.044	3	-
6	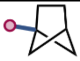		1	0.21	-	-	-	-
7	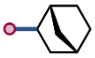		115	0.14	0.80	0.057	14	Y (<0.0001)
8	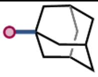		597	0.14	0.97	0.0004	21	Y (<0.0001)
9	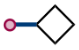		1075	0.12	0.81	<0.0001	38	Y (<0.0001)
10	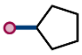		2551	0.05	0.81	0.001	52	Y (<0.0001)
11	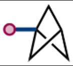		10	0.04	0.50	0.796	2	-
12	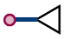		3982	0.02	0.88	0.115	55	Y (<0.0001)
13	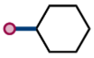		9672	-0.01	0.81	0.234	65	Y (<0.0001)
14	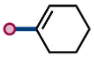		165	-0.09	0.73	0.128	18	N (0.059)
15	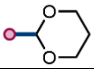		5	-0.09	0.67	0.774	-	-
16	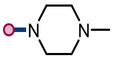		356	-0.13	1.03	0.016	24	Y (0.001)
17	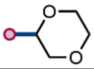		25	-0.19	0.97	0.336	5	N (0.538)
18	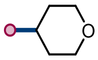		876	-0.22	0.91	<0.0001	35	Y (<0.0001)
19	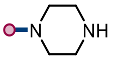		142	-0.23	1.01	0.007	17	Y (0.001)
20	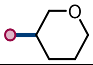		92	-0.26	0.66	0.0003	9	N (0.100)
21	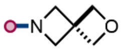		5	-0.27	0.92	0.541	3	-
22	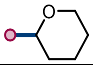		80	-0.33	0.72	<0.0001	17	Y (0.032)
23	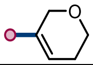		5	-0.34	0.86	0.426	2	-
24	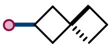		11	-0.44	0.58	0.029	-	-
25	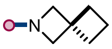		3	-0.45	0.12	0.024	-	-

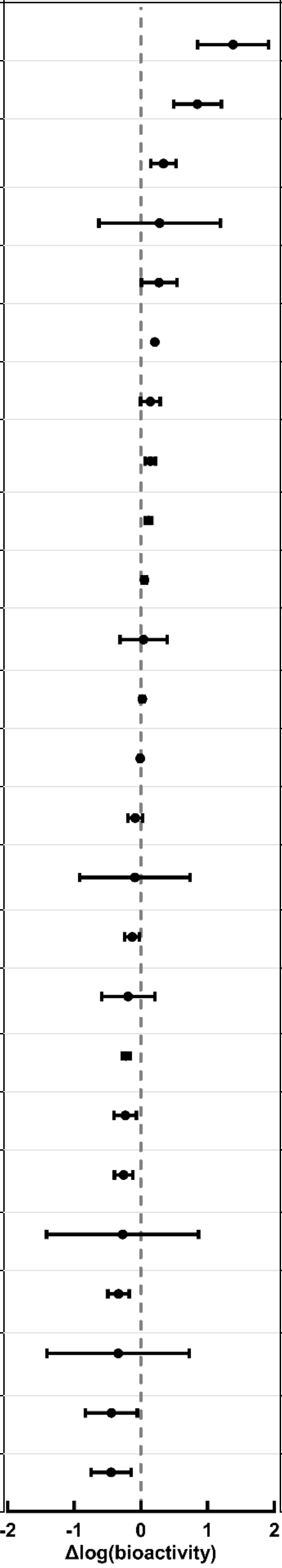

Table S2. Summary of the results obtained for the bioactivity of *para*-substituted benzene rings. Mean values are plotted with error bars representing 95% confidence intervals.[a] Mean of Δ(−log(bioactivity)); [b] A one-sample *t*-test was performed to assess whether the mean significantly differs from zero; [c] Number of protein target families as per ChEMBL classification (level 3); [d] Welch's ANOVA was used to test whether mean bioactivity changes differed significantly across target families, accounting for unequal variances.

Entry	Transformation	Mean difference (95% CI)	N	Mean[a]	SD	P value[b]	Target Families[c]	Context effects (P value)[d]
1	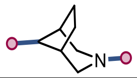	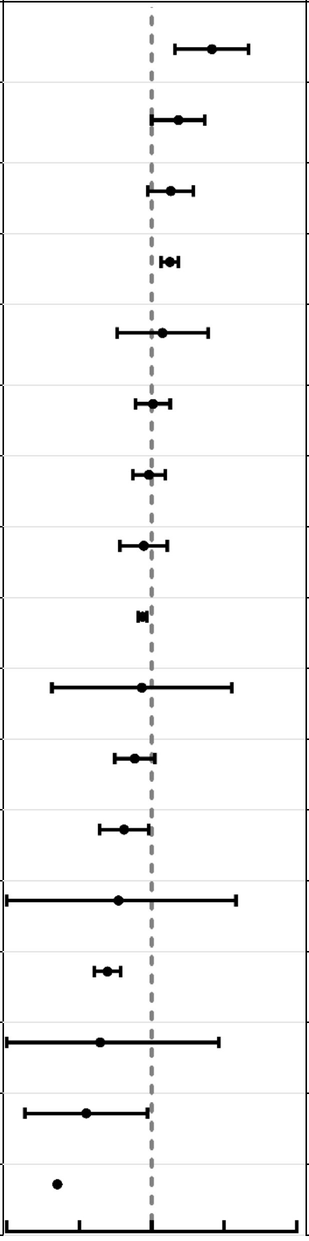	4	0.83	0.32	0.014	2	-
2	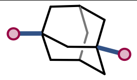		23	0.37	0.85	0.049	3	Y (0.047)
3	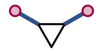		51	0.26	1.11	0.094	9	Y (<0.0001)
4	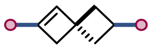		10	0.25	0.16	0.001	-	-
5	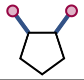		13	0.15	1.04	0.612	2	-
6	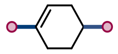		58	0.02	0.90	0.867	10	N (0.734)
7	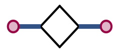		59	-0.04	0.86	0.745	9	Y (0.004)
8	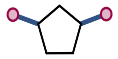		22	-0.11	0.73	0.495	6	N (0.094)
9	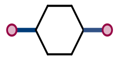		876	-0.12	0.90	<0.0001	39	Y (<0.0001)
10	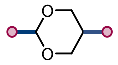		4	-0.13	0.78	0.754	-	-
11	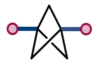		32	-0.23	0.77	0.102	4	N (0.573)
12	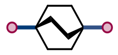		27	-0.38	0.85	0.029	5	Y (0.014)
13	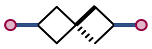		4	-0.46	1.02	0.436	1	-
14	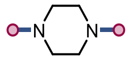		96	-0.61	0.89	<0.0001	11	N (0.210)
15	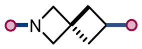		3	-0.71	0.66	0.204	1	-
16	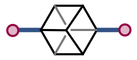		4	-0.90	0.53	0.043	1	-
17	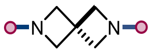		1	-1.30	-	-	1	-

Table S3. Summary of the results obtained for the bioactivity of *meta*-substituted benzene rings. Mean values are plotted with error bars representing 95% confidence intervals.[a] Mean of Δ(−log(bioactivity)); [b] A one-sample *t*-test was performed to assess whether the mean significantly differs from zero; [c] Number of protein target families as per ChEMBL classification (level 3); [d] Welch's ANOVA was used to test whether mean bioactivity changes differed significantly across target families, accounting for unequal variances.

Entry	Transformation	Mean difference (95% CI)	N	Mean[a]	SD	P value[b]	Target Families[c]	Context effects (P value)[d]
1	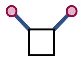		6	0.62	0.38	0.011	1	-
2	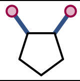		19	0.45	1.16	0.105	3	-
3	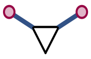		35	0.44	1.33	0.059	7	Y (<0.0001)
4	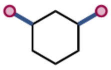		112	0.16	0.81	0.040	18	N (0.100)
5	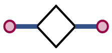		21	-0.20	0.78	0.256	4	-
6	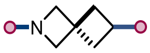		3	-0.22	0.17	0.152	-	-
7	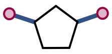		22	-0.32	0.88	0.103	5	N (0.742)
8	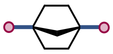		3	-0.45	0.10	0.016	1	-
9	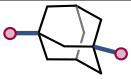		9	-1.53	3.11	0.178	2	-

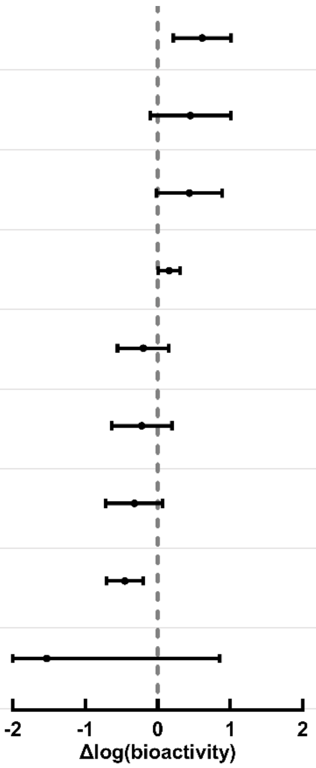

Table S4. Summary of the results obtained for the bioactivity of *ortho*-substituted benzene rings. Mean values are plotted with error bars representing 95% confidence intervals.[a] Mean of Δ(−log(bioactivity)); [b] A one-sample *t*-test was performed to assess whether the mean significantly differs from zero; [c] Number of protein target families as per ChEMBL classification (level 3); [d] Welch's ANOVA was used to test whether mean bioactivity changes differed significantly across target families, accounting for unequal variances.

Entry	Transformation	Mean difference (95% CI)	N	Mean[a]	SD	P value[b]	Target Families[c]	Context effects (P value)[d]
1	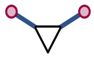		26	0.92	1.21	0.001	4	-
2	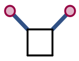		5	0.39	0.59	0.216	1	-
3	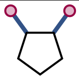		28	0.37	0.66	0.006	5	-
4	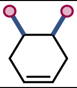		18	0.25	1.12	0.355	3	-
5	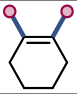		48	0.07	0.53	0.370	7	Y (0.006)
6	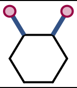		116	-0.02	1.04	0.854	13	Y (<0.0001)
7	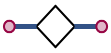		10	-0.29	1.05	0.407	2	-
8	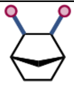		6	-0.56	1.16	0.291	2	-

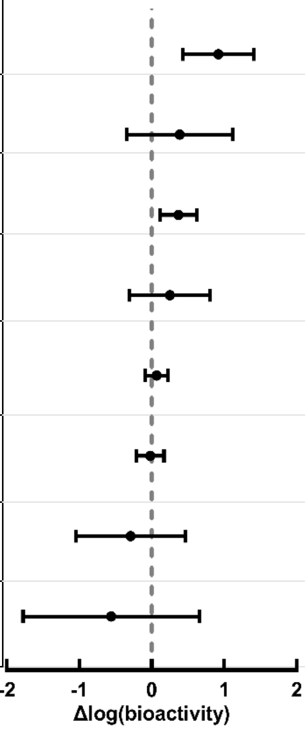

Table S5. Summary of the effect of potentially bioisosteric replacements on solubility. Mean values are plotted with error bars representing 95% confidence intervals.[a] Mean of Δ(log(solubility)); [b] A one-sample *t*-test was performed to assess whether the mean significantly differs from zero. Entries 1-9: monosubstituted benzene replacements; entries 10-12: *para*-substituted benzene replacements; entries 13-14: *meta*-substituted benzene replacements; entry 15: *ortho*-substituted benzene replacement.

Entry	Transformation	Mean difference (95% CI)	N	Mean[a]	SD	P value[b]
1	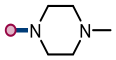	*	5	+1.09	0.73	**0.0284**
2	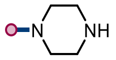		6	+1.00	1.57	0.1795
3	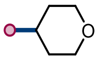	****	25	+0.99	0.75	**<0.0001**
4	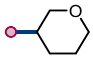		3	+0.92	0.66	0.136
5	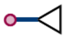	****	42	+0.63	0.90	**<0.0001**
6	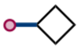		10	0.26	0.62	0.2196
7	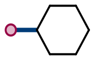		51	+0.20	0.83	0.0915
8	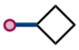		13	-0.01	0.51	0.9608
9	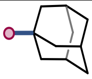		9	-0.31	0.82	0.2849
10	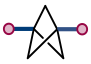	**	6	+1.20	0.54	**0.0029**
11	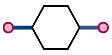	***	17	+0.46	0.47	**0.0009**
12	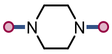		3	+0.16	0.56	0.6726
13	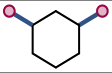		5	+0.85	1.01	0.1316
14	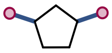	**	4	+0.77	0.17	**0.0027**
15	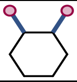	*	3	+1.12	0.36	**0.0321**

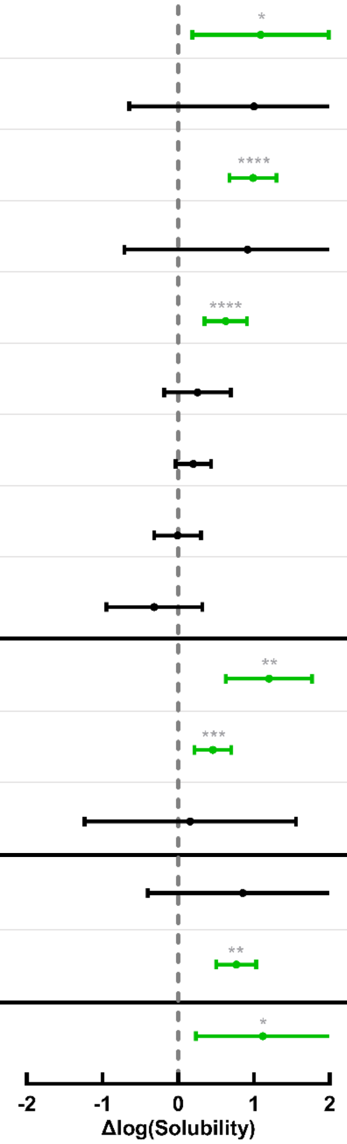

Table S6. Summary of the effect of potentially bioisosteric replacements on Cl_{int}. Mean values are plotted with error bars representing 95% confidence intervals.[a] Mean of $\Delta(\log(Cl_{int}))$; [b] A one-sample *t*-test was performed to assess whether the mean significantly differs from zero. Entries 1-7: monosubstituted benzene replacements; entries 8-10: *para*-substituted benzene replacements; entry 11: *meta*-substituted benzene replacement.

Entry	Transformation	Mean difference (95% CI)	N	Mean[a]	SD	P value[b]
1	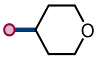		32	-0.38	0.43	**<0.0001**
2	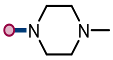		8	-0.21	0.75	0.4441
3	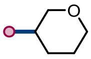		6	-0.17	0.50	0.4495
4	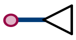		55	-0.12	0.51	0.0954
5	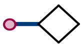		15	0.06	0.50	0.6531
6	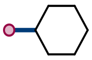		63	0.20	0.49	**0.0025**
7	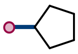		28	0.20	0.61	0.0938
8	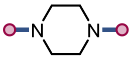		6	0.03	0.24	0.7978
9	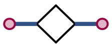		7	0.10	0.32	0.4426
10	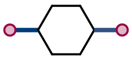		13	0.30	0.60	0.0919
11	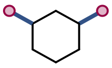		9	-0.35	0.38	**0.0238**

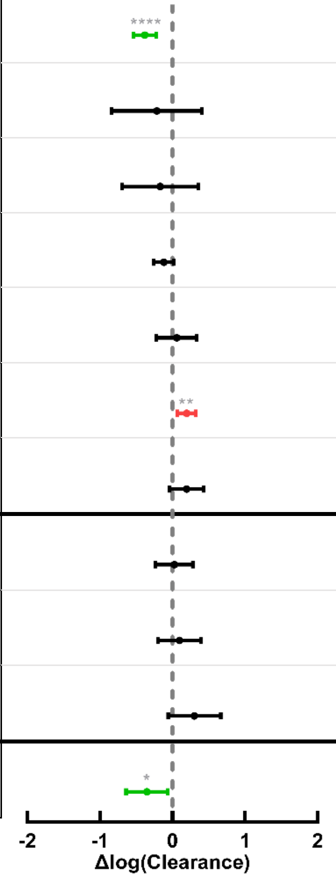

Table S7. Summary of the effect of potentially bioisosteric replacements on membrane permeability. Mean values are plotted with error bars representing 95% confidence intervals.[a] Mean of $\Delta(\log(P_{app}))$; [b] A one-sample *t*-test was performed to assess whether the mean significantly differs from zero. Entries 1-6: monosubstituted benzene replacements; entry 7: *para*-substituted benzene replacement; entry 8: *meta*-substituted benzene replacement.

Entry	Transformation	Mean difference (95% CI)	N	Mean[a]	SD	P value[b]
1	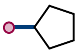		13	+0.12	0.3539	0.2303
2	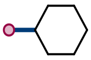		51	+0.07	0.6416	0.4237
3	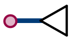		24	+0.06	0.7111	0.6940
4	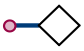		9	+0.04	0.1863	0.5532
5	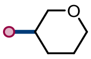		3	-0.02	0.406	0.9536
6	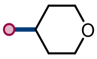		19	-0.09	0.6542	0.5499
7	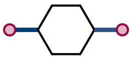		10	+0.08	0.172	0.189
8	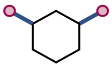		3	+0.25	0.474	0.461

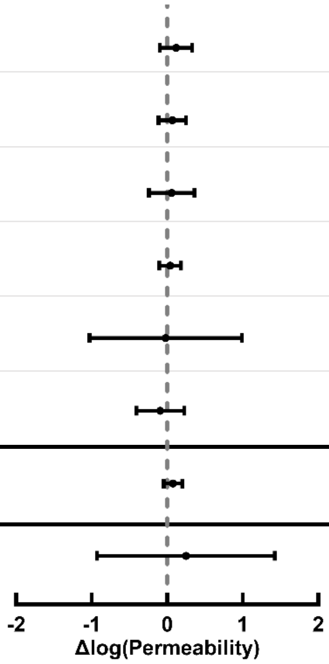

2. Analysis of the impact of stereochemistry on bioactivity

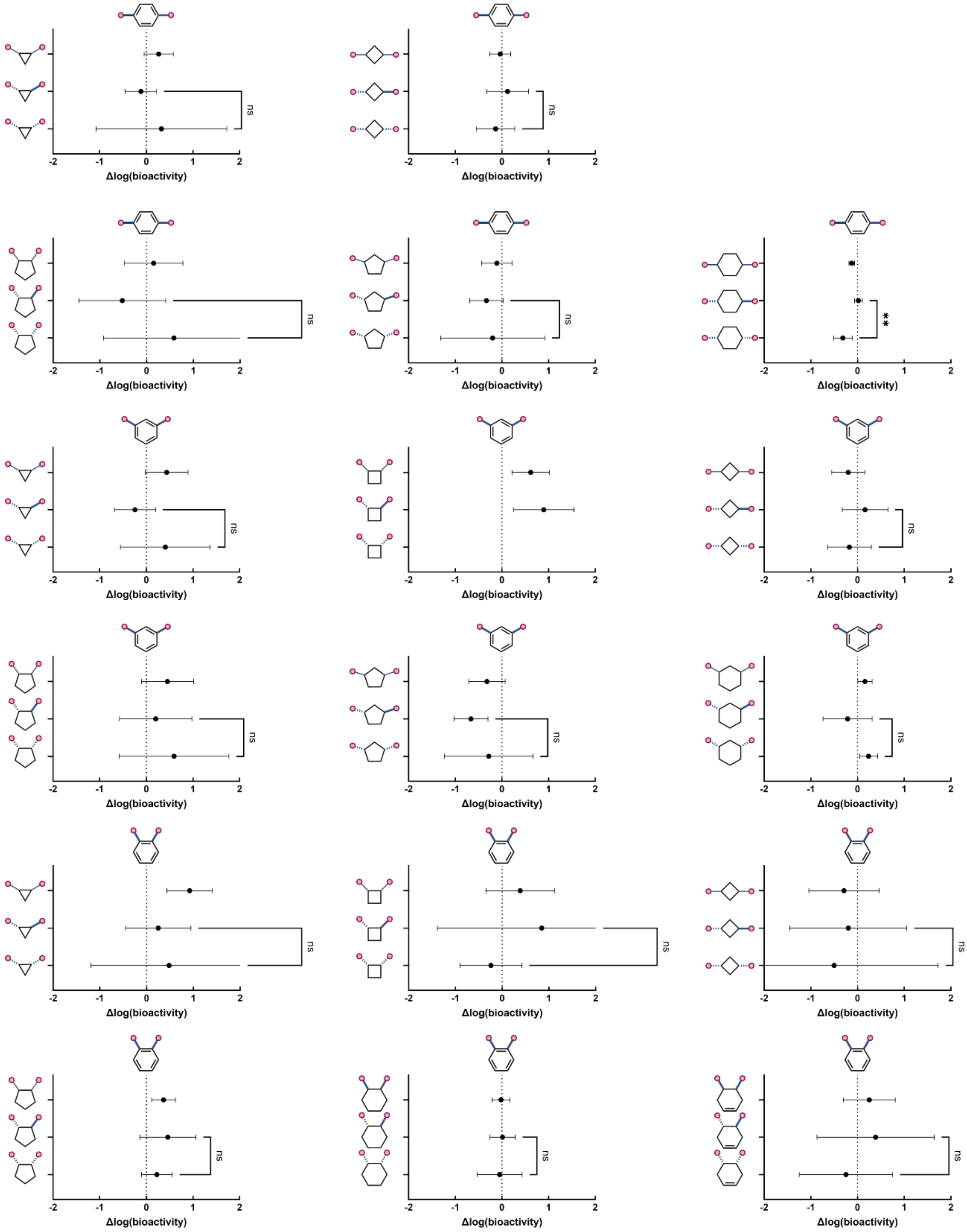

Figure S1. Impact of the potentially bioisosteric replacement of benzene with disubstituted cycloalkanes of differing stereochemistry on bioactivity. Data are presented as mean ±95% confidence interval. Welch's *t*-test was used to assess whether the mean bioactivity changes differed significantly between *cis* and *trans* isomers, accounting for unequal variances. Significance is indicated as follows: $p < 0.05$ (*), $p < 0.01$ (**).

3. Correlation analysis between compound properties and cLogP

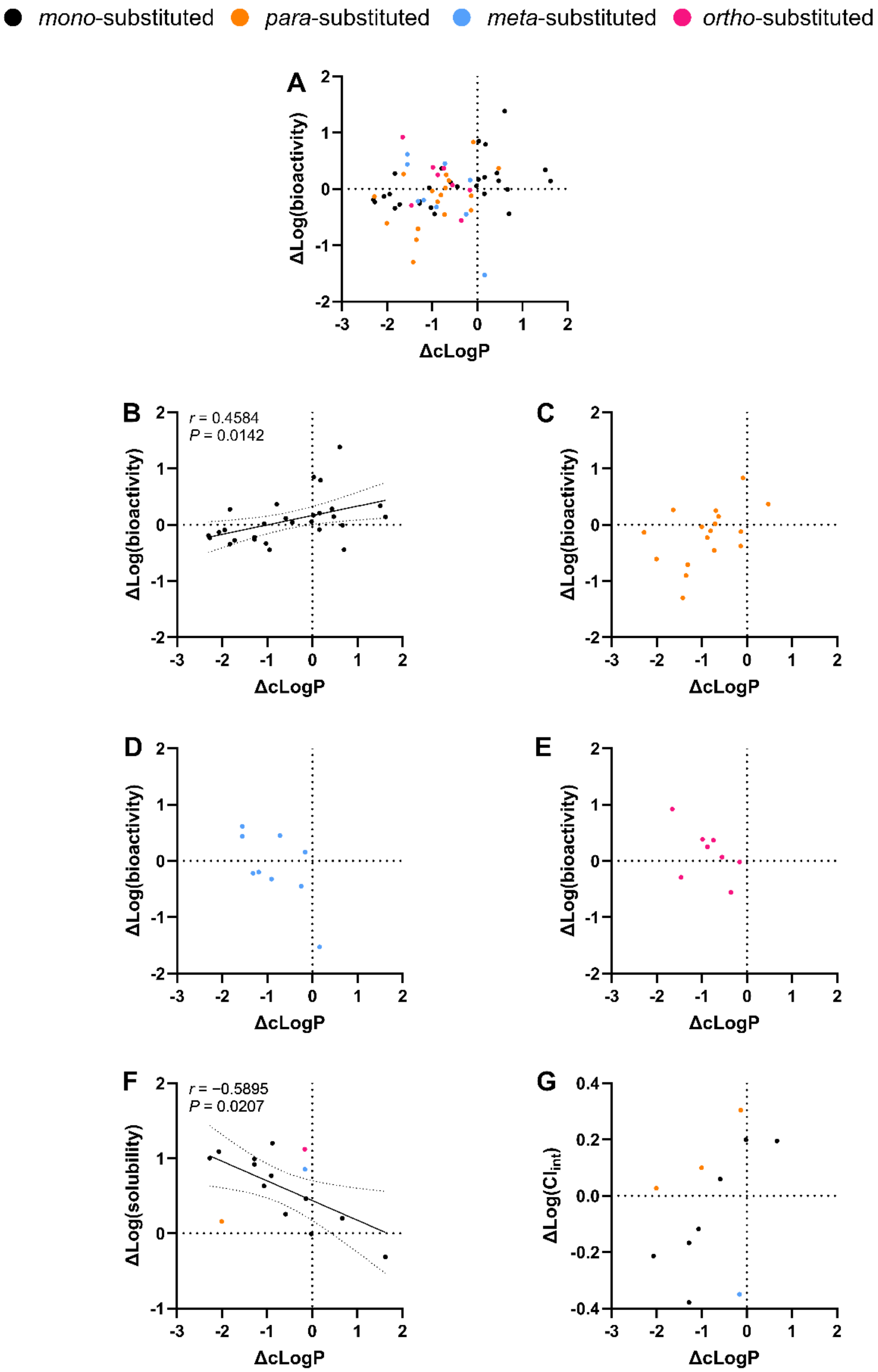

Figure S2. Correlation analysis between changes in compound properties and cLogP. Scatter plots showing the relationship between ΔLog(bioactivity) (**A**: all; **B**: *mono-;* **C**: *para-;* **D**: *meta-;* **E**: *ortho-*), ΔLog(solubility (**F**), ΔLog(Cl_{int}) (**G**) and ΔcLogP. Each point represents the average change in properties associated with a given bioisosteric replacement. Statistical significance (*P* values) and Pearson correlation coefficients (*r*) are shown in panels where P<0.05.

4. BioiSosTere Analysis and Ranking (BioSTAR) workflow user guide

Workflow Overview

The BioiSosTere Analysis and Ranking (BioSTAR) workflow enables the data-mining of a database to evaluate the impact of a specific bioisosteric replacement on molecular properties such as bioactivity, solubility, clearance, and membrane permeability. It employs KNIME as the data-mining software. The BioSTAR workflow (shown in Figure S2) can be found in KNIME Community Hub (https://hub.knime.com/).

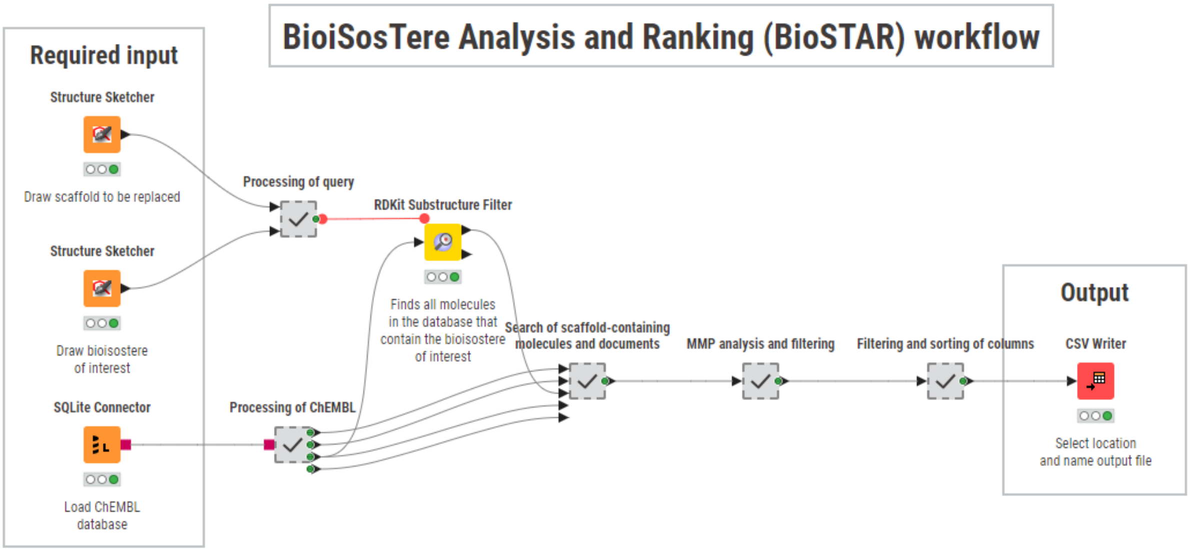

Figure S3. Appearance of the BioSTAR workflow.

Installation and Setup

System requirements:

- KNIME Analytics Platform version 5.3 or later (recommended: latest stable release).
- ChEMBL database in SQLite format (.db).
- Sufficient memory and disk space (at least 32 GB RAM recommended for large queries).

Required KNIME extensions:

Before running the workflow, ensure that the following extensions are installed:

- KNIME Chemistry Base nodes
- KNIME Javasnippet
- KNIME RDKit nodes feature
- KNIME-CDK
- Vernalis KNIME Nodes

To install extensions:

- Go to Menu → Install extensions.
- Search for each extension by name.
- Select the checkbox next to the correct package.
- Click Next and follow the prompts to complete installation.
- Restart KNIME when prompted.

Installing the Workflow:

The workflow is hosted on the KNIME Hub. To download it:

1. Visit the BioSTAR KNIME Hub page (https://hub.knime.com/s/5Ns3wwqRutwZl3y0).
2. Click "**Download**" or drag the workflow into your KNIME workspace.
3. KNIME will import the workflow into your local workspace. Open it from the KNIME Explorer panel.

First-Time Setup:

1. When you first open the workflow, KNIME may prompt you to install missing extensions. Accept and install all required extensions to ensure full functionality.
2. Configure the "**SQLite Connector**" node to point to your local ChEMBL database file. Then, run the "**Database Processing**" metanode to prepare the data. This step may take several minutes but only needs to be completed once.
3. After setup, check the workflow for any red (error) nodes. Ensure that all nodes and components load correctly before proceeding.

Running BioSTAR

Once the first-time setup is complete, follow these steps to run the workflow:

1. In the first "**Structure Sketcher**" node, enter the structure you wish to replace (e.g., benzene). You can either draw the structure or input it as a SMILES string.
2. In the second "**Structure Sketcher**" node, provide the bioisostere of interest (e.g., bicyclo[1.1.1]pentane). As before, the structure can be drawn or entered as a SMILES code.
3. Set up the "**CSV Writer**" node by choosing a destination folder and filename for the results.
4. Run the entire workflow by selecting "**Execute all**" from the toolbar.
5. Open the generated CSV file using DataWarrior or another chemical database viewer and extract the information required. For large result sets, substructure filtering may be required.

Output Details

The workflow output (CSV file) contains the following information, in successive columns:

Column name	Description	Example
**Transformation**	Molecular transformation between the matched molecular pairs	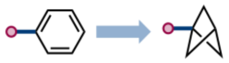 [1*]c1ccccc1>>[1*]C12CC(C1)C2
**ID (L)**	ChEMBL ID number for the parent molecule	2515670
**ID (R)**	ChEMBL ID number for the molecule containing the bioisostere of interest	2506013
**Left Fragment**	Scaffold replaced	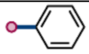 [1*]c1ccccc1
**Right Fragment**	Bioisostere of interest	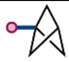 [1*]C12CC(C1)C2
**Assay ID**	ChEMBL assay ID number	2060626
**Parameter**	Molecular property measured	IC_{50}
**Relation**	Relation between parameter and value (=, < or >)	=
**Value (L)**	Value of the molecular property measured for the parent compound	400
**Value (R)**	Value of the molecular property measured for the molecule containing the bioisostere of interest	120
**Units**	Unit of measure of the molecular property measured	nM
**pXC50 (L)**	$-\log_{10}$(Value(L))	6.40
**pXC50 (R)**	$-\log_{10}$(Value(R))	6.92
**pXC50 (R-L)**	pXC50 (R) - pXC50 (L)	0.52
**SMILES (L)**	Structure of the parent compound	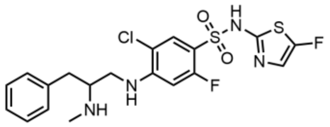 CN[CH](CNc1cc(F)c(S(=O)(=O)Nc2ncc(F)s2)cc1Cl)Cc1ccccc1
**SMILES (R)**	Structure of the molecule containing the bioisostere of interest	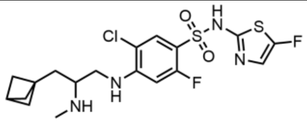 CN[CH](CNc1cc(F)c(S(=O)(=O)Nc2ncc(F)s2)cc1Cl)CC12CC(C1)C2
**ChEMBL ID**	ChEMBL document ID number	CHEMBL4706775
**Document type**	Data source (publication or patent)	Publication
**doi/patent_id**	DOI or patent number of the primary data source	10.1021/acsmedchemlett.1c00218

Customization

The BioSTAR workflow may be used to analyse databases other than ChEMBL after appropriate modification of the “Database processing” node.

Successive searches may be performed if the workflow is equipped with a “loop” functionality.

Troubleshooting

**Issue**	**Solution**
The structure sketcher shows the error: “The dialog cannot be opened for the following reason: “*Cannot invoke “org.openscience.cdk (…) because “container” is null.*“	Replace the node by a new “Structure Sketcher” node.
Workflow takes a very long time.	The search may be too broad or the computer limited in capacity. Narrow down the structure search and/or increase the computer power.
A node from a KNIME extension shows an error message.	Check for updates and install if available.
